# Photoinduced host-to-guest electron transfer in a self-assembled coordination cage†

**DOI:** 10.1039/d2qo01339h

**Published:** 2022-08-24

**Authors:** Sudhakar Ganta, Jan-Hendrik Borter, Christoph Drechsler, Julian J. Holstein, Dirk Schwarzer, Guido H. Clever

**Affiliations:** Department of Chemistry and Chemical Biology, TU Dortmund University Otto-Hahn Straße 6 44227 Dortmund Germany guido.clever@tu-dortmund.de; Max-Planck-Institute for Multidisciplinary Sciences Am Fassberg 11 37077 Göttingen Germany

## Abstract

A [Pd_2_L_4_] coordination cage, assembled from electron-rich phenothiazine-based ligands and encapsulating an electron-deficient anthraquinone-based disulfonate guest, is reported. Upon excitation at 400 nm, transient absorption spectroscopy unveils photoinduced electron transfer from the host's chromophores to the guest, as indicated by characteristic spectral features assigned to the oxidized donor and reduced acceptor. The structure of the host–guest complex was characterized by NMR spectroscopy, mass spectrometry and single-crystal X-ray analysis. Spectroelectrochemical experiments and DFT calculations both agree with the proposed light-induced charge separation. A kinetic analysis of the involved charge transfer channels reveals, besides a guest-independent LMCT path, 44% efficiency for the host–guest charge transfer (HGCT).

## Abstract 2

Light–powered host–guest charge transfer (HGCT) is shown for a coordination cage based on electron-rich phenothiazines, containing an anthraquinone acceptor as guest. Transient absorption spectroscopy and spectroelectrochemistry data is presented.

## Graphical abstract

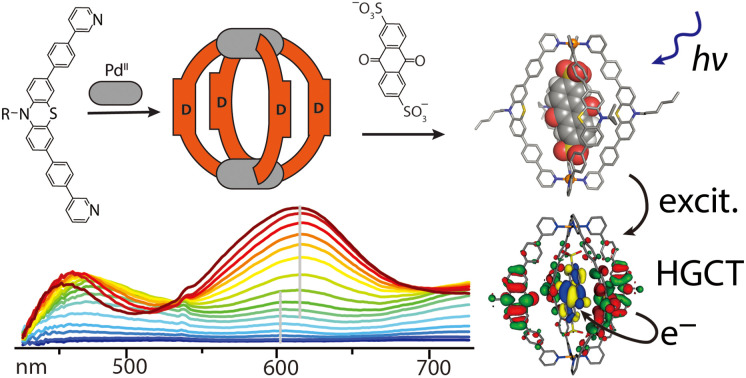

## Introduction

In nature, photosynthesis is the fundamental reaction cascade that transforms light into chemical energy, involving light-induced charge separation within precisely arranged chromophore assemblies. Taking this as an inspiration, the quest to build efficient artificial molecular photosystems that harvest solar energy and transform it into an electrical potential difference or power the formation of high-energy fuel compounds is a key scientific challenge.^1–4^ In this respect, organic and inorganic donor–acceptor (D–A) systems have gained considerable attention due to their ability to promote charge separation upon irradiation.^5–8^ A wide variety of potent approaches to design efficient D–A systems has been introduced over the years.^6,9,10^ In most reported discrete systems, donor and acceptor moieties are connected through some kind of linker by conventional covalent chemistry and examined for light-triggered charge transfer between excited donor and acceptor. However, connecting multiple donors/acceptors *via* covalent bonds under precise control over their optimal distance and spatial orientation can be synthetically challenging and tedious. Recent progress in supramolecular chemistry provides alternative approaches to conventional multistep synthesis by connecting donor- and acceptor-functionalized building blocks through noncovalent or coordinative interactions, allowing the simple and high yielding assembly of D–A combinations in a modular fashion with determined stoichiometry and spatial arrangement.^11–13^ Such supramolecular D–A systems have been explored to act as artificial photosynthetic systems that harvest light energy, as electron-transfer systems, hydrogen evolution catalysts, and further photo-redox-functional devices.^9,13–19^

Self-assembled coordination cages,^20–24^ featuring a confined yet accessible inner space, have been of interest as a nanoscopic container for guest encapsulation,^25–27^ as a molecular transport vehicle,^28,29^ as a stabilizer of reactive compounds^30^ and as a supramolecular catalytic center.^31^ Furthermore, the interaction between cages and guest molecules in terms of photophysical processes was also explored before.^32–40^ Self-assembled supramolecular systems represent an ideal platform for realizing photoinduced electron transfer (PET) processes due to the possibilities to arrange donor and acceptor functionalities in short through-space distance, achieving maximum local concentration, and defined orientation.^18,35,36,38,41–44^

The choice of the D–A pair and the spatial arrangement of the D and A moieties play crucial roles to effectively achieve a charge separated state with long lifetime, a prerequisite for application of such systems as a basis for active materials in photovoltaic devices. As donor components, electron-rich aromatics such as phenothiazines (PTZ), arylamines as well as thiophene and carbazole derivatives are frequently chosen. Anthraquinones (AQ), benzothiadiazoles, squaraines and various cyano- and keto-functionalized heterocycles have been employed as complementary partners to construct a D–A system.^45^ As a popular combination, PTZ and AQ derivatives are often implemented as D–A pairs owing to their suitable redox potentials and HOMO–LUMO energy gap characteristics.^46,47^

Previously, we introduced a self-assembly approach to amalgamate PTZ- and AQ-based building blocks in a D–A system based on interpenetrated [Pd_4_L_8_] cage assemblies.^48,49^ We first demonstrated that the self-assembly of banana-shaped PTZ- or AQ-based bismonodentate pyridyl ligands (named D or A, respectively) with Pd(ii) cations yielded homoleptic catenated double-cages [3BF_4_@Pd_4_L_8_]^5+^ (L = D or A).^48,50^ Further, we showed that the equimolar mixture of these ligands (D : A = 1 : 1) with Pd(ii) ions resulted in a statistical combination of mixed-ligand interpenetrated cages [Pd_4_D_*m*_A_8−*m*_]^8+^ (*m* = 8…0), containing all possible stoichiometries and stereoisomeric combinations. By far most of the components of the resulting mixture were mixed-ligand double-cages, containing at least one donor or one acceptor component, that contain densely packed PTZ and AQ functionalities in the immediate vicinity within the highly entangled supramolecular assemblies. Upon irradiation with light of 385 nm wavelength, electron transfer from the excited state phenothiazine donor onto the anthraquinone acceptor to obtain a charge-separated state could be unambiguously corroborated by a combination of transient absorption experiments in the UV-Vis and IR spectral regions. However, the fact that the examined samples in this study consisted of a statistical mixture of mixed-ligand cages hampered interpretation of the photophysical kinetics of the decisive species and restricts the understanding of structure–function relationships and the establishment of design principles for next-generation D–A systems.

Here, we report an alternative approach towards the rational design of discrete D–A systems composed of a metallosupramolecular host, assembled from electron-rich donor ligands, and an electron-deficient acceptor compound, serving as a guest. We elucidate the formation and structure of the donor-functionalized coordination cage and its host–guest complex by NMR spectroscopy, mass spectrometry, and single crystal X-ray analysis. The photophysical and electrochemical properties of the host–guest complex are described. Transient absorption experiments show that the donor-functionalized cage is able to transfer electrons to the incarcerated guest molecule within the host–guest assembly upon irradiation with light of proper wavelength.

## Results and discussion

### Design of the system

**Scheme 1:**
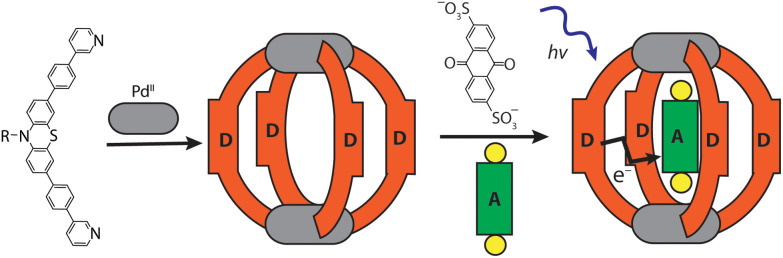
Self-assembly of donor cage **1** and host–guest complex [**G1**@**1**]^2+^.

**Fig. 1:**
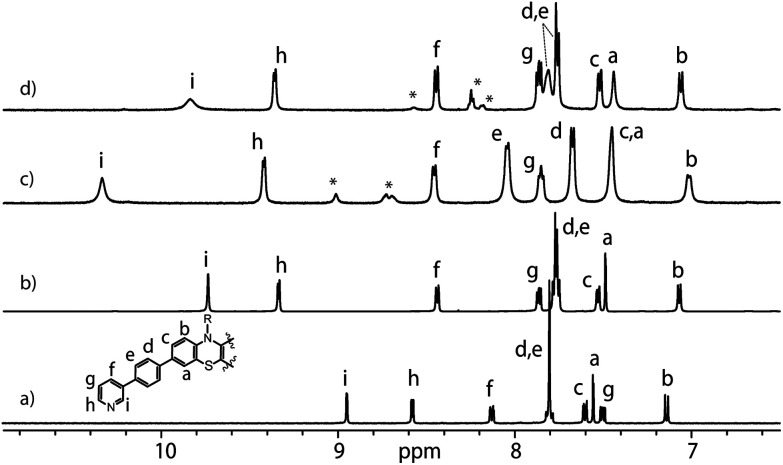
Partial ^1^H NMR spectra (500 MHz, DMSO-*d*_6_, 298 K) of **L** (a), donor-functionalized cage **1** (b), host–guest assemblies [**G1**@**1**]^2+^ (c) and [**G3**@**1**]^3+^ (d). Signals of **G1** and **G3** are indicated by *.

**Fig. 2:**
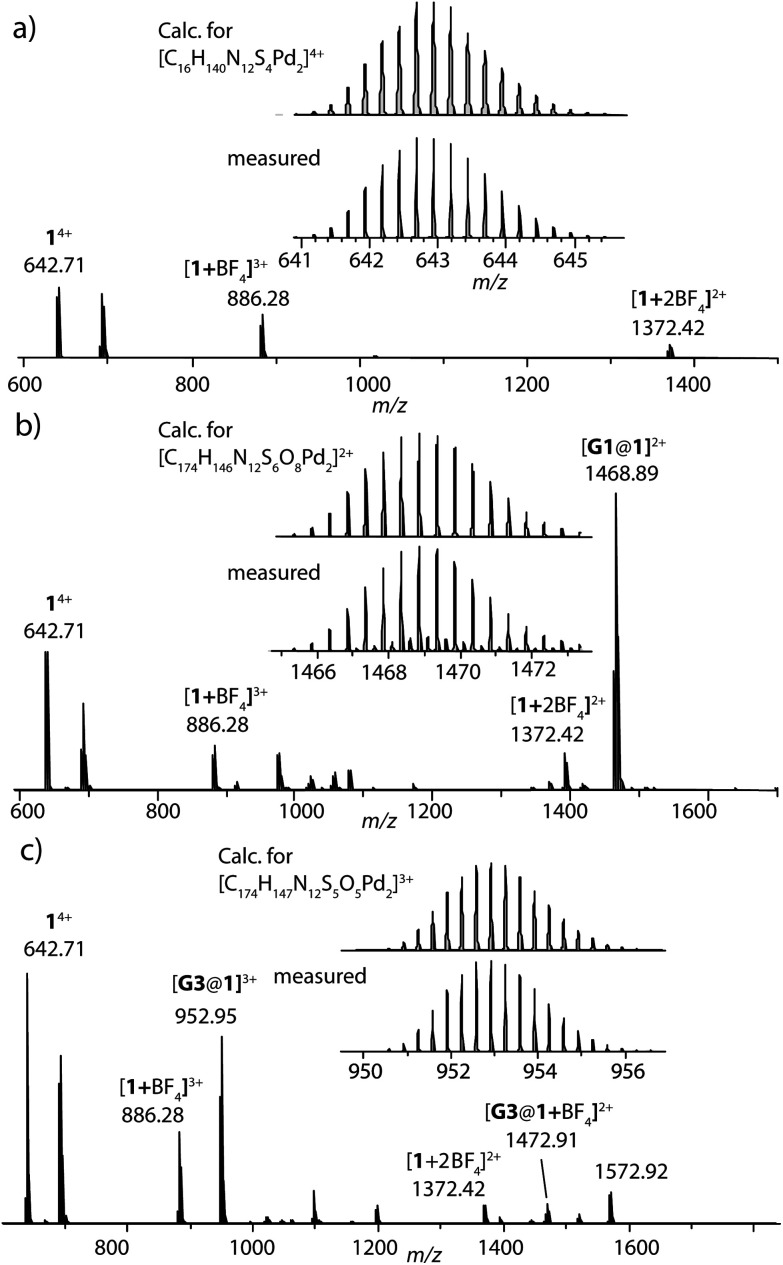
HR-ESI mass spectra of donor cage **1** (a), host–guest assemblies [**G1**@**1**]^2+^ (b) and [**G3**@**1**]^3+^ (c).

Ligand **L** was synthesized by Suzuki–Miyaura coupling of the dibromo derivative of a hexyl-substituted phenothiazine (PTZ) backbone with a boronic ester of phenyl pyridine in good yield (Scheme S1 and Fig. S1–S5†). The ligand is comparable to a PTZ ligand, previously reported by us.^50^ However, the ethynyl linker was replaced with a 1,4-phenylene bridge which resulted in the increase of the ligand's length (in terms of distance between coordinating nitrogens) by about 3 Å. Self-assembly of the ligand with palladium precursor [Pd(CH_3_CN)_4_](BF_4_)_2_ in 2 : 1 ratio resulted in quantitative formation of desired donor cage [Pd_2_**L**_4_](BF_4_)_4_**1** in DMSO solution (Scheme 1). The formation of the cage was unambiguously confirmed by ^1^H NMR spectroscopy (Fig. 1, and Fig. S6–S9†) and high-resolution mass spectrometry analysis (Fig. 2a, and Fig. S10†). In the ^1^H NMR spectrum of cage **1**, signals of the pyridine rings (H_i_, H_h_, H_f_, and H_g_) were downfield shifted compared to the free ligand, characteristic of the metal-mediated self-assembly. Signals corresponding to di-, tri-, and tetra-cationic complexes of the cage **1** with a differing number of BF_4_^−^ counter anions were identified in the ESI mass spectrum (Fig. 2). ^1^H DOSY NMR also supported the formation of a single species with a hydrodynamic radius of 13.6 Å (Fig. S26†). All these studies confirm the successful assembly of four electron-rich ligands into a 3-dimensional cage **1**. Next, cage **1** was explored in encapsulating electron-poor guest molecules.

### Host–guest chemistry

Anthraquinones (AQs) are found in important compound classes such as natural pigments and redox carriers, anticancer drugs, and pesticides. They are also extensively studied in the context of redox flow batteries and photocatalysis, owing to their advantageous acceptor properties, reversible redox behaviour, and fast electrochemical kinetics. In this study, sodium salts of anthraquinone-2,6-disulfonate (**G1**), anthraquinone-2,7-disulfonate (**G2**), and anthraquinone-2-sulfonate (**G3**) were examined as suitable guest molecules for the donor-functionalized host **1** in DMSO. From ^1^H NMR studies it was observed that only **G1** and **G3** were encapsulated inside cage **1** (Fig. 1), but not **G2** (Fig. S13†). The size and shape of **G2** are probably not compatible with the cavity of cage **1**.^51^

The addition of one equivalent of **G1** or **G3** to a solution of cage **1** resulted in the formation of host–guest assemblies [**G1**@**1**]^2+^ and [**G3**@**1**]^3+^, respectively, within 5 min at room temperature, as confirmed by analysing the corresponding ^1^H NMR spectra (Fig. 1). Compared to the parental species, a significant change was observed in the ^1^H NMR spectrum of [**G1**@**1**]^2+^. Particularly, protons pointing towards the cavity (*i.e.* H_i_ and H_e_) of the host are most affected after the addition of the guest molecule **G1**. Proton H_i_, which points inside the cavity, is downfield shifted by about 0.64 ppm, suggesting strong interactions with the guest's sulfonate groups. In contrast, phenothiazine backbone signals (H_a_, H_b_ and H_c_) along with signal H_d_ were found somewhat upfield shifted. In the case of [**G3**@**1**]^3+^, only proton H_i_ is downfield shifted by about 0.1 ppm and broadened compared to free cage **1**. A competitive ^1^H NMR experiment was performed by combining **G1**, **G2**, and **G3** in equimolar ratio (1 : 1 : 1) with cage **1** (1 equiv.) to check guest preference and selective uptake of **G1** was observed (Fig. S24†).

Careful stoichiometric titration of guests **G1** or **G3** to host **1** revealed fast exchange kinetics on the NMR timescale, resulting in a single set of signals for both host and guest molecules upon guest addition (Fig. S19, and S20†). An estimation of the binding constant for **G1** and **G3** was derived from the titration experiments using the Bindfit online tool.^52,53^ As expected, the binding constant of dianionic **G1** (≈ 1.16 × 10^5^ M^−1^) is superior to monoanionic **G3** (≈ 140 M^−1^; Fig. S21, S22 and compare S50†). DOSY analysis suggested that the diffusion coefficient and size of the host are not much affected by encapsulation of the guest molecule inside the cavity (Fig. S26–S28†). Association between host and guest was also observed in the ESI-MS results. The ESI mass spectra of the host–guest assemblies show strong signals at *m*/*z* = 1469 and 953, assigned to cations [**G1**@**1**]^2+^ and [**G3**@**1**]^3+^, respectively (Fig. 2). Hence, all spectroscopic results indicate that the host–guest complexes of the anthraquinone acceptors inside the phenothiazine donor cages are the predominant species in DMSO solution at millimolar concentrations.

### Crystal structure analysis

**Fig. 3:**
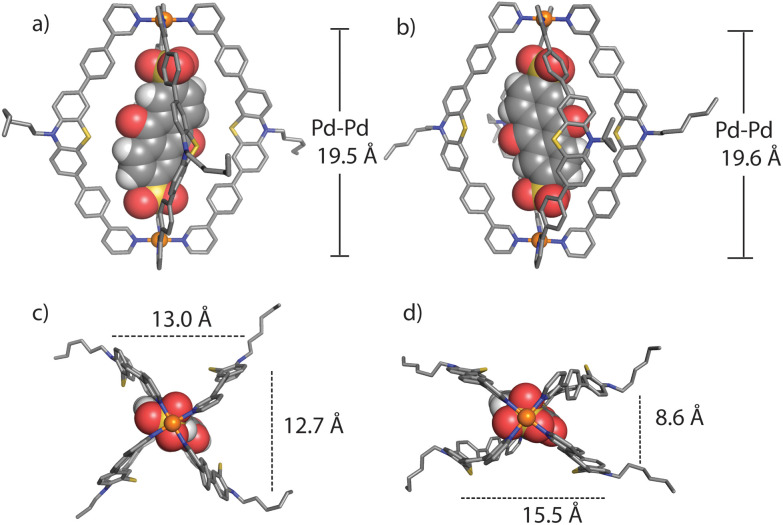
Two conformational isomers in the crystal structure of the host–guest assembly [**G1**@**1**]^2+^: conformer A (**HG-A**, left) and conformer B (**HG-B**, right), in side view along the Pd–Pd axis (a and b), and downward the Pd–Pd axis (c and d) (hydrogen substituents, solvent molecules and other **G1** anions are omitted for clarity).

We succeeded in obtaining single-crystal X-ray diffraction data for ligand **L** (Fig. S29†) and host–guest complex [**G1**@**1**]^2+^ (Fig. 3). Slow diffusion of 1,4-dioxane into a DMSO solution of [**G1**@**1**]^2+^(with an excess of **G1**) over a period of four weeks resulted in tiny needle-shaped single crystals that proofed suitable for synchrotron X-ray diffraction. The host–guest assembly crystallized in triclinic form, showing space group *P*1̄ (2). The asymmetric unit contains two host–guest assemblies [**G1**@**1**]^2+^ besides two unbound **G1** dianions and fourteen DMSO molecules (Fig. S30–S36†). The Pd⋯Pd distance within the cage is about 19.6 Å. The central cavity of the cage is occupied by one guest molecule. The inward pointing α-protons of the pyridine units are in close contact to the oxygen atoms of sulfonate groups (the shortest distance between S–O⋯H–C is 2.27 Å). The ligands’ phenylene linkers also seem to help stabilizing the guest inside the cavity with similar interactions (the shortest distance between S–O⋯H–C is 2.37 Å). The AQ moiety is surrounded by four PTZ-based ligands of the cage assemblies, however, the guest's aromatic system does not show any significant direct noncovalent interactions with the host structure in both conformers found in the X-ray structure result. The minimum and maximum distances between the centroids of the PTZ and AQ aromatic rings are about 7.6 and 7.9 Å, respectively. One of the two cage conformers in the asymmetric unit is more extended, almost obeying a fourfold symmetry when inspected downwards the Pd–Pd axis (Fig. 3a and c), while the other conformer shows a more folded shape (Fig. 3b and d). In the latter case, the AQ major plane is positioned between the concave faces of two of the bent PTZ units while the other two PTZ units are facing towards the AQ unit with their edges in a slightly angled way. Overall, the acceptor AQ moieties of both host–guest conformers are surrounded by the four donor PTZ units in direct spatial proximity while not directly engaging in any close π–π-interaction. Owing to the fact that the NMR spectra of the host–guest assemblies do not show any guest-induced signal splitting of the host protons, free and rapid rotation of the guest inside the host can be anticipated in solution, most probably in a way that the guest's sulfonate-sulfonate axis coincides with the host's Pd–Pd axis.

### Theoretical studies

**Fig. 4:**
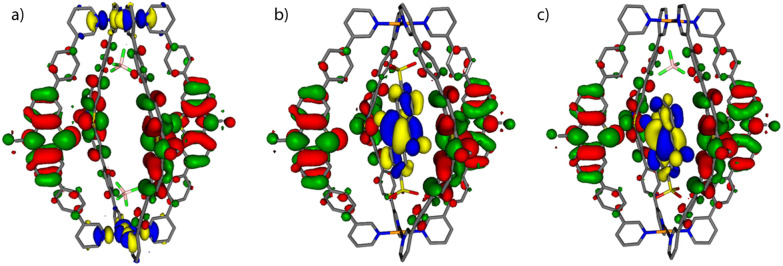
Frontier molecular orbital visualization of (a) donor-functionalized cage **1** (containing two BF_4_^−^ counter anions), and host–guest assemblies (b) [**G1**@**1**]^2+^ and (c) [**G3**@**1** + BF_4_]^2+^. The HOMOs are represented in red-green, LUMOs in yellow-blue.

Molecular models of the cage with only two counter anions [**1** + 2BF_4_]^2+^ and the host–guest assemblies [**G1**@**1**]^2+^ and [**G3**@**1** + BF_4_]^2+^ were first optimized on B3LYP/def2-SVP level and the electronic properties were then calculated with B3LYP/def2-TZVP and implicit solvation (see ESI† for details).^54^ As can be seen in Fig. 4, the fourfold degenerate HOMOs are spread over the PTZ moieties of the ligands **L** for every system. But while for [**1** + 2BF_4_]^2+^ the twofold degenerate LUMOs are found at the palladium(ii) centers, the LUMO for each host–guest assembly is spread over the acceptor guests **G1** and **G3**, respectively. The calculated energy gaps between HOMO and LUMO are 1.95 eV for [**G1**@**1**]^2+^ and 1.97 eV for [**G3**@**1** + BF_4_]^2+^, while it is 2.92 eV for [**1** + 2BF_4_]^2+^ (Table S2†).

### UV-Visible spectroscopy studies

Absorption spectra of ligand **L**, cage **1**, guests **G1** and **G3**, and the host–guest assemblies were recorded in DMSO solution at ambient temperatures (Fig. S39†). The absorption spectra revealed maxima at 295 nm and 372 nm for **L**; 292 nm and 370 nm for cage **1**; 284 nm and 333 for **G1**, 276 nm and 330 nm for **G3**. The absorption spectra of the host–guest assemblies are merely a sum of their components, indicating the absence of strong electronic communication between the donor and acceptor moieties at the ground state.

### Cyclic voltammetry

**Fig. 5:**
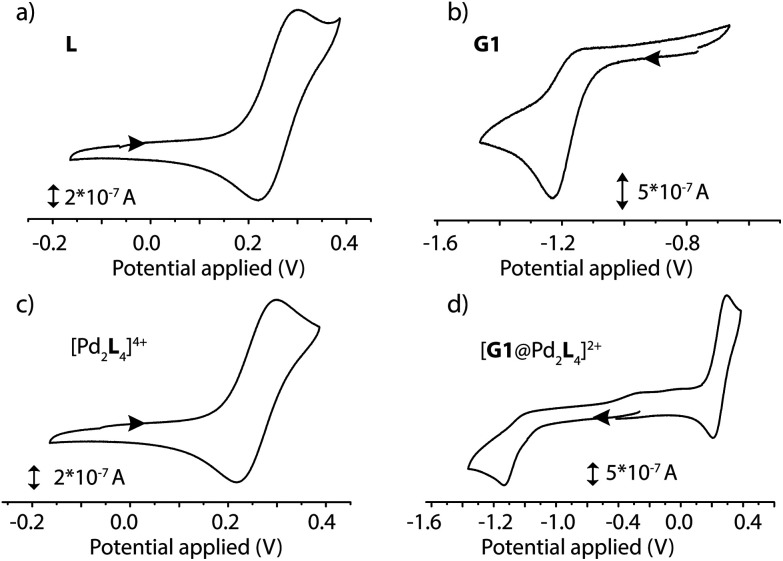
Cyclic voltammograms of (a) ligand **L** (1.4 mM in DMSO), (b) guest **G1** (0.35 mM in 0.1 M [NBu_4_][PF_6_] DMSO), (c) cage **1** (0.7 mM in 0.1 M [NBu_4_][PF_6_] DMSO) and (d) host–guest complex [**G1**@**1**]^2+^, (0.7 mM in 0.1 M [NBu_4_][PF_6_] DMSO), recorded at a glassy carbon working electrode against a Ag/AgNO_3_ reference electrode at 298 K. Scan rate 100 mV s^−1^.

The electrochemical properties of the D–A assemblies and their individual components were studied by cyclic voltammetry in DMSO solution at 298 K, with 0.1 M of TBAPF_6_ serving as the electrolyte at a sweep rate of 100 mV s^−1^ against an Ag/AgNO_3_ reference electrode, in a glovebox under Ar atmosphere. The resulting voltammograms are depicted in Fig. 5 and Fig. S41.† Ligand **L** shows a quasi-reversible wave for the PTZ moiety with an oxidation signal at 0.30 V and a reduction at about 0.22 V potential. Donor-functionalized cage **1** displays similar oxidation and reduction potentials for the PTZ moieties (0.30 and 0.22 V, respectively). The oxidation and reduction signals for the PTZ moieties in the host–guest assembly [**G1**@**1**]^2+^ are shifted to slightly lower positive potentials (0.29 and 0.21 V, respectively; Δ*E*_ox_ = 0.01 V, and Δ*E*_red_ = 0.01 V). Free guest **G1** has its reduction and oxidation potentials at about −1.23 and −1.17 V, respectively. In the host–guest assembly [**G1**@**1**]^2+^, the reduction potential (about −1.14 V) for the acceptor moiety is shifted towards a more positive potential in comparison to free **G1** (Δ*E*_red_ = 0.09 V).

### Spectroelectrochemistry

**Fig. 6:**
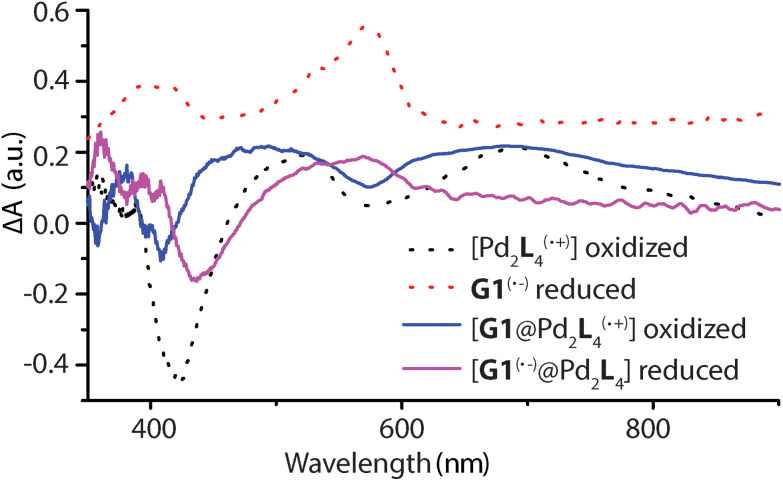
Electrochemically generated difference absorption spectra for the oxidized [Pd_2_**L**^(^˙^+)^_4_], [**G1**@Pd_2_**L**^(^˙^+)^_4_] and reduced **G1**^(^˙^−)^, [**G1**^(^˙^−)^@Pd_2_**L**_4_] species (number of oxidized **L** units and overall charges not specified in formulas). UV-Vis spectra of samples recorded during cyclic voltammetry with a scan rate of 0.1 V s^−1^, Pt gauze as working electrode and Ag/AgNO_3_ as reference electrode.

Spectroelectrochemical measurements of **L**, **1**, **G1**, and [**G1**@**1**] were performed in a thin cell (1 mm optical path length) containing a Pt gauze and a reference electrode (Ag/AgNO_3_) in combination with a diode array spectrophotometer. The resulting difference spectra are depicted in Fig. 6 and Fig. S42–S45.† The spectrum of oxidized **L**^(^˙^+)^ and [Pd_2_**L**_4_^(^˙^+)^] are similar, and a sharp absorption peak around 518 nm and a very broad peak around 691 nm is apparent in both cases. The reduced guest **G1** revealed a broad signal around 574 nm. In the case of host–guest assembly [**G1**@**1**], the resulting spectra shows both signals for the oxidized donor and the reduced acceptor at respective voltages, similar to the free cage and guest at comparable potentials. Thus, donors and acceptor were found to maintain their characteristic electrochemical behaviours in the self-assembled host–guest complex and did not show any significant redox interplay in the ground state.

### Transient absorption spectroscopy

Femtosecond pump–probe spectroscopy was applied to get insight into the dynamics following photoexcitation of the bare donor ligand **L** as well as the host cage [Pd_2_**L**_4_]^4+^, with and without the presence of equimolar amounts of guest molecules **G1** or **G3**. All compounds were investigated in DMSO solution. They were excited at *λ*_exc_ = 400 nm (where **G1** and **G3** do not absorb) and probed in the UV-Vis at 390–730 nm using a white-light continuum.

Pump pulse-induced difference spectra for the free donor ligand **L** (see Fig. S46†) indicate population of a long-lived S_1_ state with maxima at 450 and 613 nm and a minimum at around 500 nm, produced by superposition of excited state absorption and stimulated emission. The stimulated emission component exhibits a dynamic Stokes shift of 1000 cm^−1^ within several ps. The relaxed excited state lifetime of >2 ns is consistent with a bright stationary fluorescence and similar to other phenothiazine compounds.^55^

**Fig. 7:**
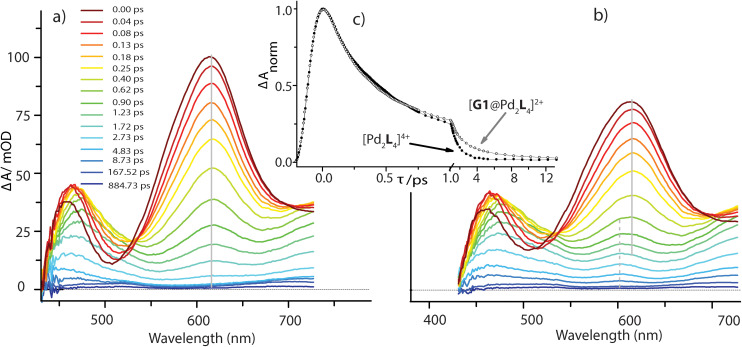
Transient absorption spectra of the bare host cage [Pd_2_**L**_4_]^4+^ (a) and in the presence of equimolar amounts of **G1** (b), following excitation at 400 nm. For both measurements the kinetic traces at a probe wavelength of 613 nm are compared in panel (c) (note that the scaling of time axis changes at 1.0 ps).

In the [Pd_2_**L**_4_]^4+^ cage, the steady-state fluorescence of the phenothiazine ligand is completely quenched. This observation is consistent with the temporal evolution of the [Pd_2_**L**_4_]^4+^ transients presented in Fig. 7a. Directly after 400 nm excitation, a strong positive band at 613 nm characteristic for the S_1_ state of the donor ligand (hereafter denoted as **L**^S1^) appears, however, its lifetime is only ∼0.7 ps. The minimum at 505 nm, indicating stimulated emission from **L**^S1^, is less pronounced and gets even weaker and more red-shifted with time compared to the free ligand transients (Fig. S46†). In addition, above 700 nm the absorption slightly increases during the donor's S_1_ decay and only at times >0.4 ps starts to drop with a characteristic time of ∼1 ps. This is consistent with the formation of a short-lived radical cation **L**^(^˙^+)^, exhibiting absorption bands at 500 and 700 nm (*cf.* spectra in Fig. 6). In our previous study on a [3BF_4_@Pd_4_L_8_]^5+^ double cage, we have seen similar efficient fluorescence quenching of a related PTZ ligand derivative L due to a ligand-to-metal charge transfer (LMCT) to the bound Pd(ii), followed by immediate back electron transfer (BET).^48,49^Fig. 7a suggests a similar mechanism operating in [Pd_2_**L**_4_]^4+^, for which photo-induced LMCT leads to the intermediate [Pd^II^Pd^I^**L**_3_**L**^(^˙^+)^]^4+^. In Fig. 7a, the **L**^(^˙^+)^ characteristic absorption at 500 nm appears rather weak because (i) it is superimposed by the stimulated emission of the donor's S_1_ state and (ii) BET is so fast that the [Pd^II^**L**_3_Pd^I^**L**^(^˙^+)^]^4+^ population is always low.

The spectral evolution of the photo-excited [Pd_2_**L**_4_]^4+^ cage was found to be not affected by addition of the electron acceptor **G3** to the cage at the same concentration used as for the host alone (0.35 mM in cage = 0.09 mM per PTZ; Fig. S47†). Given the low binding constant of **G3**, we suspected that the host–guest equilibrium is too far on the left side at the used concentrations which could be confirmed by an NMR experiment (Fig. S50†). However, the presence of equimolar amounts of stronger binding guest **G1** leads to subtle but significant changes in the excited state dynamics (see Fig. 7b) compared to the bare host cage. Whereas for the latter, the 613 nm band assigned to **L**^S1^ stays constant at this wavelength while decaying (*cf.* vertical gray line in Fig. 7a), Fig. 7b indicates a blue-shift of 13 nm starting at pump probe delays ≳1 ps (dashed grey line). We attribute the new band emerging at 600 nm to the reduced guest **G1**^(^˙^−)^ being formed by electron transfer from one of the phenothiazine ligands in the coordination cage. The difference in peak position compared to the electrochemically generated spectrum of reduced **G1** (*λ*_max_ = 575 nm, see Fig. 6) may be caused by the electric field inside the cage, associated with the proximity of two positively charge Pd^2+^ and one **L**^(^˙^+)^ ions. Formation of **G1**^(^˙^−)^ by host-to-guest charge transfer (HGCT) is also supported by a closer inspection of the decay kinetics of the 613 nm band. The grey data points in Fig. 7c show that with **G1** the decay of this band is first faster and then turns over to a slower decay than without the guest (black points). Also for other probe wavelengths, time traces were found to be altered upon encapsulation of **G1** (Fig. S49†). The initial faster decay suggests that the presence of **G1** opens up an additional reaction channel towards HGCT, accelerating depopulation of the donor's S_1_ state. The following slower decay indicates that BET between the [Pd_2_**L**_4_^(^˙^+)^]^4+^-**G1**^(^˙^−)^ couple is significantly slower than for the [Pd^II^**L**_3_Pd^I^**L**^(^˙^+)^]^4+^ intermediate.

**Fig. 8:**
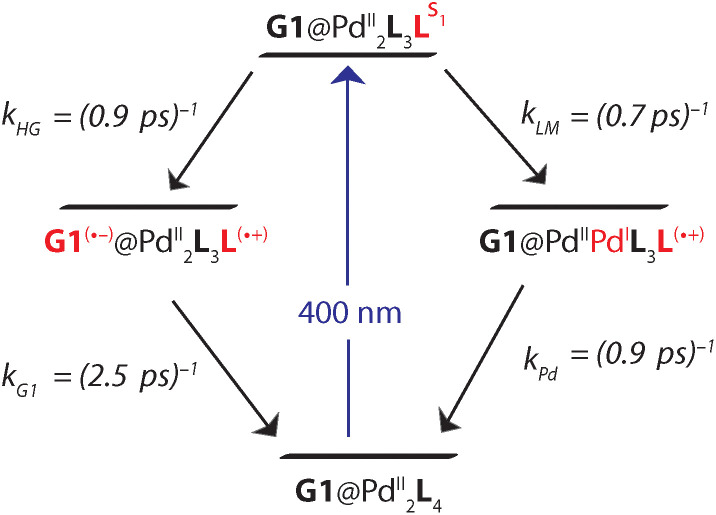
Charge transfer channels of the photoexcited [Pd_2_**L**_4_]^4+^ cage. The right branch represents LMCT with subsequent BET and is independent on whether **G1** is present or not. The left branch corresponds to HGCT followed by BET in the presence of **G1**.

Based on the reaction scheme depicted in Fig. 8, which involves the two identified charge transfer channels, we calculated time dependent absorption profiles of those species emerging in the transient spectra (**L**^S1^, **L**^(^˙^+)^, and **G1**^(^˙^−)^). For four selected wavelengths these were fitted to measured time traces using rate constants and relative absorption cross sections *σ*_*i*_(*λ*) of the three species *i* as adjustable parameters (for details see the ESI†). In a first step, the kinetics of the bare host cage was fitted (*i.e.* omitting the left branch of the reaction scheme in Fig. 8) yielding the rate constants *k*_LM_ = (0.7 ± 0.1 ps)^−1^ and *k*_Pd_ = (0.9 ± 0.2 ps)^−1^ for LMCT and corresponding BET, respectively, as well as the relative cross sections *σ*(**L**^S1^, *λ*) and *σ*(**L**^(^˙^+)^, *λ*) (Fig. S48a†). Then, in a second step, the kinetics of [**G1**@Pd_2_**L**_4_] was fitted allowing for HGCT competing with LMCT, which yields *k*_HG_ = (0.9 ± 0.2 ps)^−1^ and *k*_G1_ = (2.5 ± 0.3 ps)^−1^ for HGCT and corresponding BET, respectively, as well as the relative cross section *σ*(**G1**^(^˙^−)^, *λ*) (Fig. S48b†). The relative yield of *Φ*_HG_ = *k*_HG_/(*k*_HG_ + *k*_LM_) = 44% shows that a significant fraction of excited state population undergoes host-to-guest electron transfer. This analysis assumes that the LMCT dynamics do not change when **G1** is taken up by the cage. This is supported by the absorption spectrum of [**G1**@Pd_2_**L**_4_] which is just the sum of the spectra of **1** and **G1**, indicating that there is no special interaction between host and guest that alters the electronic structure of the cage. In particular, there is no close π–π-interaction between host and guest aromatic parts. The main driving force for establishing decent host–guest affinity is the perfect arrangement of the guest's sulfonate groups close to both of the cage's inner Pd(pyridine)_4_ faces, thereby replacing loosely bound BF_4_^−^ counter anions. Also, the crystal structure of the host–guest complex and DFT-based geometry-optimized models of the cage without guest are almost superimposable, *i.e.* the Pd–Pd distance is similar in both cases.

## Conclusions

Herein, a self-assembled donor-functionalized cage based on electron-rich PTZ moieties is reported. The cage is capable of encapsulating electron-deficient acceptor molecules such as anthraquinone-2,6-disulfonate and anthraquinone-2-sulfonate. The crystal structure of one of the host–guest assemblies [**G1**@**1**]^2+^ confirmed the presence of the guest in the central cavity of the cage. Theoretical studies of the host–guest assemblies suggested that HOMOs are spread over the donor cage components while the LUMOs are localized on the guest molecules with an energy gap of about 188 kJ mol^−1^. Spectroelectrochemistry results revealed the characteristics of the radical ions of the donor and acceptor components to be maintained in the host–guest assembly and the absence of electronic communication between donor and acceptor in the ground state. However, the femtosecond transient UV-Vis absorption spectroscopy unambiguously shows the formation of a cage-based PTZ radical cation and a guest (**G1**) radical anion upon excitation of the host–guest assembly. Thus, we have demonstrated that the donor cage built from electron-rich PTZ moieties is proficient in transferring electrons to the incarcerated acceptor **G1** guest. The rational design of supramolecular donor–acceptor assemblies that promote photoinduced electron transfer will be of interest in the field of organic photovoltaics, given that modern self-assembly strategies allow to create complex and anisotropic molecular materials with potential to control the morphology of photo-active layers. Furthermore, photo-redox catalytic applications can benefit from host–guest chemistry *via* tuning substrate affinity and exchange kinetics with respect to the excited state life-time of a catalytic nanoconfinement.

## Author contributions

S. Ganta and G. H. Clever conceived and designed the study. S. Ganta performed the synthesis and characterization of the materials. J. J. Holstein performed the X-ray structures analysis. C. Drechsler conducted the computational studies. J.-H. Borter and D. Schwarzer performed and analyzed femtosecond pump–probe spectroscopy experiments. S. Ganta wrote the initial draft and all authors reviewed and edited the paper.

## Conflicts of interest

There are no conflicts to declare.

## Supplementary Material

QO-009-D2QO01339H-s001

QO-009-D2QO01339H-s002
