# Supplementary material for: Photoinduced host-to-guest electron transfer in a self-assembled coordination cage
Source: Org Chem Front. 2022 Aug 24;9(20):5485–93. doi: 10.1039/d2qo01339h (PMC9552185; doi:10.1039/d2qo01339h)
Supplement: QO-009-D2QO01339H-s001 [file QO-009-D2QO01339H-s001.pdf]

Electronic Supplementary Material (ESI) for Organic Chemistry Frontiers.
This journal is © the Partner Organisations 2022

Photoinduced Host-to-Guest Electron Transfer in a Self-Assembled Coordination Cage

Sudhakar Ganta,[1] Jan-Hendrik Borter,[2] Christoph Drechsler,[1] Julian J. Holstein,[1] Dirk Schwarzer,[2] Guido. H. Clever[1*]

[1] Department of Chemistry and Chemical Biology, TU Dortmund University, Otto-Hahn Straße 6, 44227, Dortmund, Germany

[2] Max-Planck-Institute for Multidisciplinary Sciences, Am Fassberg 11, 37077 Göttingen, Germany

Contents

1) Experimental Section.....2
1.1 Materials and methods.....2
1.2 Experimental procedures.....3
2) Synthesis.....3
2.1 Ligand.....3
2.2 Cage 1.....6
2.3 Host-guest supramolecular donor-acceptor systems:.....9
2.3.1 $[G1@1]^{2+}$:.....10
2.3.2 G2@1:.....11
2.3.3 $[G3@1]^{3+}$:.....11
2.3.4 ^{1}H NMR titration experiments:.....14
2.3.5 Binding constant.....16
2.3.6 Guest selectivity by cage 1.....17
2.4 DOSY NMR studies:.....18
3) Crystal structure.....20
3.1 Table S1. Crystal data and structure refinement for ligand L, $[G1@1]^{2+}$.....20
3.2 Crystal structure of L (sg6).....21
3.3 Crystal structure of $[G1@1]^{2+}$ (sg88y_8).....22
4) Theoretical studies.....29
4.1 Optimized structures.....29
4.2 HOMO-LUMO diagrams.....30
4.3 Table S2. Calculated HOMO-LUMO energies.....30
5) UV-Vis studies.....31
6) Fluorescence studies.....31
7) Cyclic voltammetry studies.....32
7.1.1 Experimental setup.....32
8) Spectroelectrochemistry studies.....34

8.1 Experimental setup 34
9) Transient absorption studies 39
9.1 Transient UV/vis spectra 39
9.2 Kinetic model 40
10) ^{1}H NMR spectra at lower concentration 42
11) References 42

1) Experimental Section

1.1 Materials and methods

Unless otherwise stated, all chemicals were obtained from commercial sources and used as received. NMR spectroscopic data was measured on the spectrometers Bruker AV 500 Avance NEO and AV 600 Avance III HD. For ^{1}H, chemical shifts were calibrated to the solvent lock signal. Proton signals were assigned with the aid of 2D NMR spectra. ^{1}H DOSY NMR spectra were recorded with a dstebpgp3s pulse sequence with diffusion delays D20 of 0.06-0.10 s and gradient powers P30 of 800 to 2000 µs. Electrospray ionization (ESI) mass spectra were recorded on a Bruker timsTOF Mass Spectrometer. UV-Vis spectra were recorded on a DAD HP-8453 UV-Vis spectrometer. Cuvette path length 1 mm, wavelength: 250 nm – 800 nm, step size: 1 nm.

Single-crystal X-ray diffraction data of ligand **L** were collected on a Bruker d8 venture diffractometer an Incoatec Iµs 3.0 microfocussed CuK$_\alpha$ source. Due to very thin plate-shaped crystals of supramolecular coordination cage [**G1@1**]$^{2+}$, the analysis was hampered by the limited scattering power of the samples not allowing us to reach the desired atomic resolution using in-house equipment. Gaining detailed structural insight required cryogenic crystal handling and highly brilliant synchrotron radiation. Hence, diffraction data of most of supramolecular assembly [**G1@1**]$^{2+}$ was collected at macromolecular synchrotron beamline P11, PETRA III, DESY. Disorder in ligand side chains, counterions and solvent molecules required carefully adapted macromolecular refinement protocols employing geometrical restraint dictionaries, similarity restraints and restraints for anisotropic displacement parameters (ADPs).

Geometry optimized models of structures were constructed using Wavefunction SPARTAN′18 and first optimized on semiempirical PM6 level of theory without constraints. Further, they were optimized on B3LYP/def2-SVP level, then the energy calculations were run at B3LYP/def2-TZVP.

The transient UV-Vis -pump-probe setup was described before.[1,2] Briefly, a a Ti:sapphire based oscillator/regenerative amplifier system (Solstice Ace, Spectra Physics) producing 35 fs laser pulses at 800 nm was used to create ~0.5 µJ pump pulses at 400 nm by 2nd harmonic generation in a BBO crystal. A probe white light continuum (WLC) was generated by focusing a small portion of the 800 nm beam (pulse energy 3 µJ) into a 4 mm CaF2 crystal. The pump-probe time delay was adjusted with a computer-controlled translation stage (M-415.DG, Physik Instrumente). For every laser shot about 50% of the WLC energy was used to record a reference spectrum. The other half was for probing pump pulse-induced changes in the spectrum by superimposing both beams at the center of the sample cell (UV quartz cuvette, optical path 2 mm, Starna). A synchronized chopper blocked every second pump pulse to determine difference spectra with and without the pump pulse. The relative plane of polarization of pump and probe light was adjusted to 54.7°. Both probe and reference spectra were recorded by individual spectrographs each equipped with a 256-element linear diode array. The covered spectral range was 350-730 nm. All measurements were performed with stirred

DMSO solutions at ligand concentrations of 0.35 mM. The data were corrected for shifts of the baseline (determined at negative pump probe delays) and wavelength-dependent temporal shifts due to group delay differences.

1.2 Experimental procedures

Where necessary, experiments were performed under nitrogen atmosphere using standard Schlenk techniques. Chemicals and standard solvents were purchased from Sigma Aldrich, Acros Organics, Carl Roth, TCI Europe, VWR, ABCR and used as received, if not mentioned differently. Dry solvents were purchased or purified and dried over absorbent-filled columns on a GS-Systems solvent purification system (SPS). Reactions were monitored with thin layer chromatography (TLC) using silica coated aluminium plates (Merck, silica 60, fluorescence indicator F254, thickness 0.25 mm). For column chromatography, silica (Merck, silica 60, 0.02–0.063 mesh ASTM) was used as the stationary phase.

2) Synthesis

2.1 Ligand

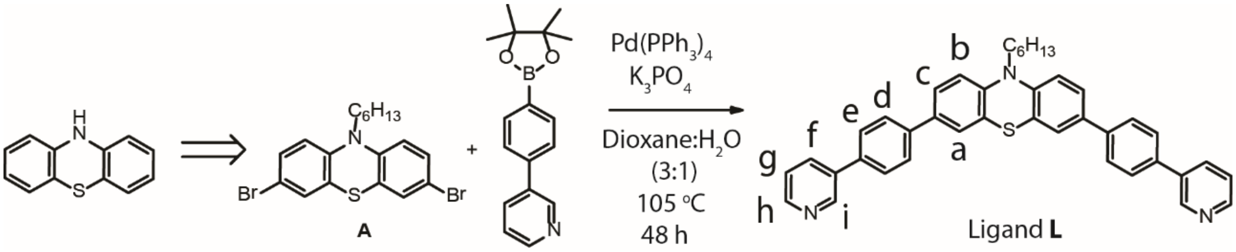

Scheme S1. Synthesis of ligand, **L**.

A mixture of 3,7-dibromo-10-hexyl-10H-phenothiazine **A**[3] and (300 mg, 0.683 mmol, 1.0 equiv.), 3-(4-pyridyl)phenylboronic acid pinacol ester (576 mg, 2.02 mmol, 3.0 eq.) and Na_2CO_3 (360 mg, 3.4 mmol, 5.0 equiiv.) was suspended in a 20 mL mixture of 1,4-dioxane/H_2O (3:1). The mixture was degassed and $Pd(PPh_3)_4$ (100 mg, 0.07 mmol, 0.1 equiv.) was added. The mixture was stirred for 48 h at 105 °C. Ethyl acetate (50 mL), and water (10 mL) were added to the mixture and the organic phase was washed with water (3 x 50 mL), dried over $MgSO_4$, filtrated and the solvent was evaporated under reduced pressure. The crude residue was purified by flash chromatography on silica gel (pentane: ethyl acetate = 9:1 to 1:1) to give the product as a yellow solid (260 mg, 0.444 mmol, 65%).

^{1}H NMR (500 MHz, $CDCl_3$): δ [ppm] = 8.90 (d, J = 2.5 Hz, 2 H), 8.60 (dd, J = 5.3 Hz, 2 H), 7.93 (dt, J = 7.9 Hz, 2 H), 7.67- 7.64 (m, 8 H), 7.45-7.44 (m, 4 H), 7.39 (dd, J = 2.9 Hz, 2 H), 6.96 (d, J = 9.0 Hz, 2 H), 3.92 (t, J = 7.2 Hz, 2 H), 1.92-1.86 (m, 2 H), 1.51-1.47 (m, 2 H), 1.36-1.33 (m, 4 H), 0.90 (t, J = 7.1 Hz, 3 H).

^{13}C NMR (126 MHz, 298 K, $CDCl_3$) δ [ppm] = 146.94, 146.69, 138.82, 135.33, 135.08, 133.54, 133.51, 126.47, 126.08, 124.90, 124.71, 123.76, 122.72, 114.54, 47.33, 30.45, 25.81, 25.65, 21.58, 12.97 (one carbon signal overlapping).

^{1}H NMR (500 MHz, DMSO-d_6): δ [ppm] = 8.95 (d, J = 1.8 Hz, 2 H), 8.56 (dd, J = 3.0 Hz, 2 H), 8.14-8.12 (m, 2 H), 7.82-7.75 (m, 8 H), 7.60 (dd, J = 6.1 Hz, 2 H), 7.55 (d, J = 2.2 Hz, 2 H), 7.50 (ddd, J = 8.0 Hz, 2 H), 7.14 (d, J = 8.5 Hz, 2 H), 3.96 (t, J = 6.9 Hz, 2 H),1.75 (q, J = 7.5 Hz, 2 H), 1.44 (q, J = 7.0 Hz, 2 H), 1.32-1.22 (m, 4 H), 0.85 (t, J = 7.0 Hz, 3 H).

ESI-HRMS $[C_{40}H_{35}N_3S+H]^+$: found: 590.2582; calc.: 590.2630

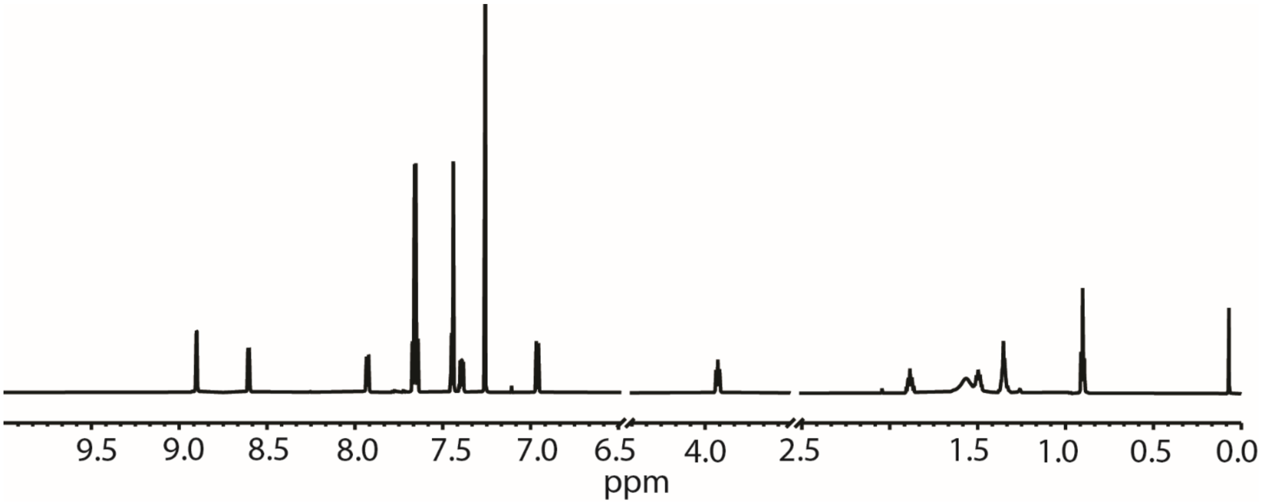

Figure S1: ^{1}H NMR spectrum (500 MHz, 298K, $CDCl_3$) of **L**.

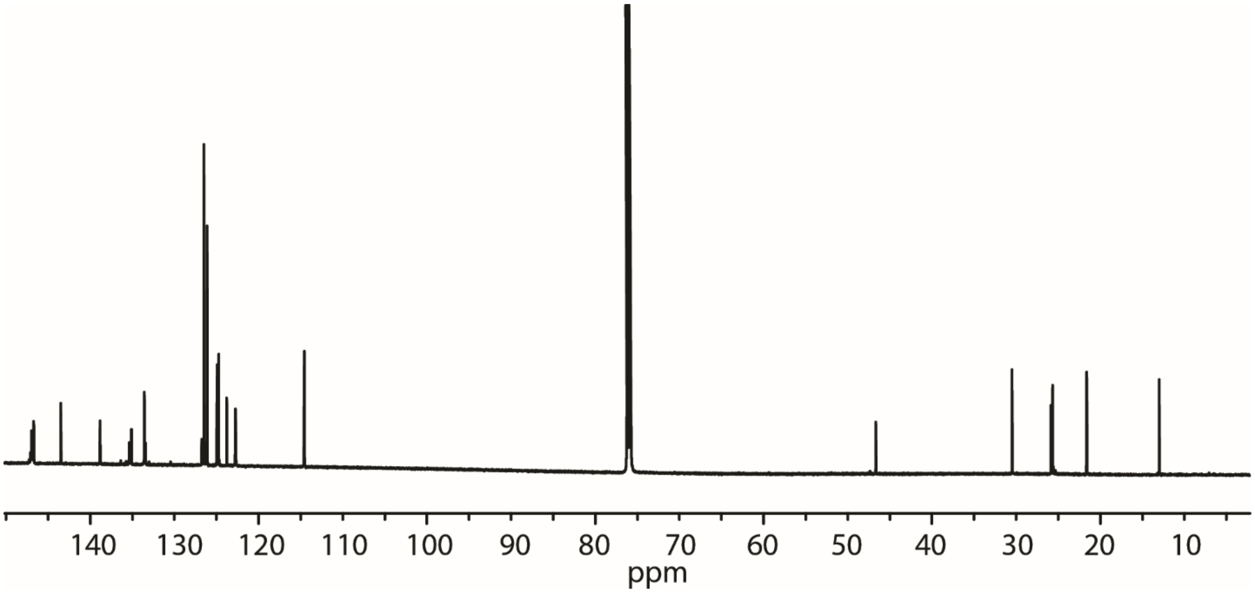

Figure S2: ^{13}C NMR spectrum (125 MHz, 298K, $CDCl_3$) of **L**.

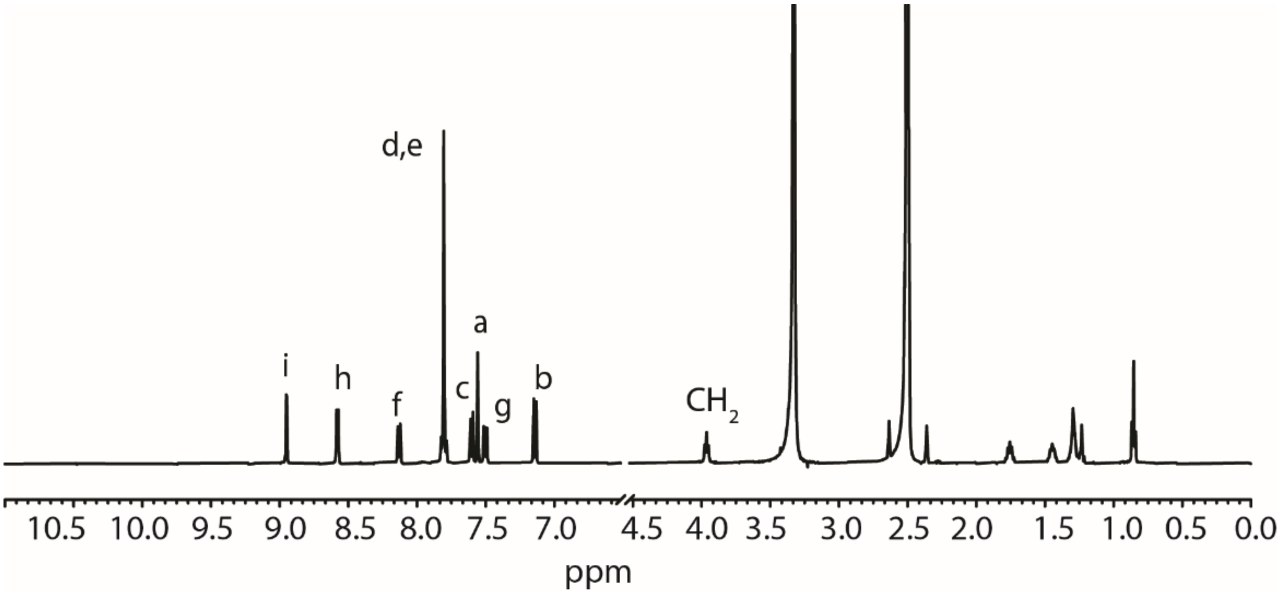

Figure S3: ^{1}H NMR spectrum (500 MHz, 298K, DMSO-d_6) of **L**.

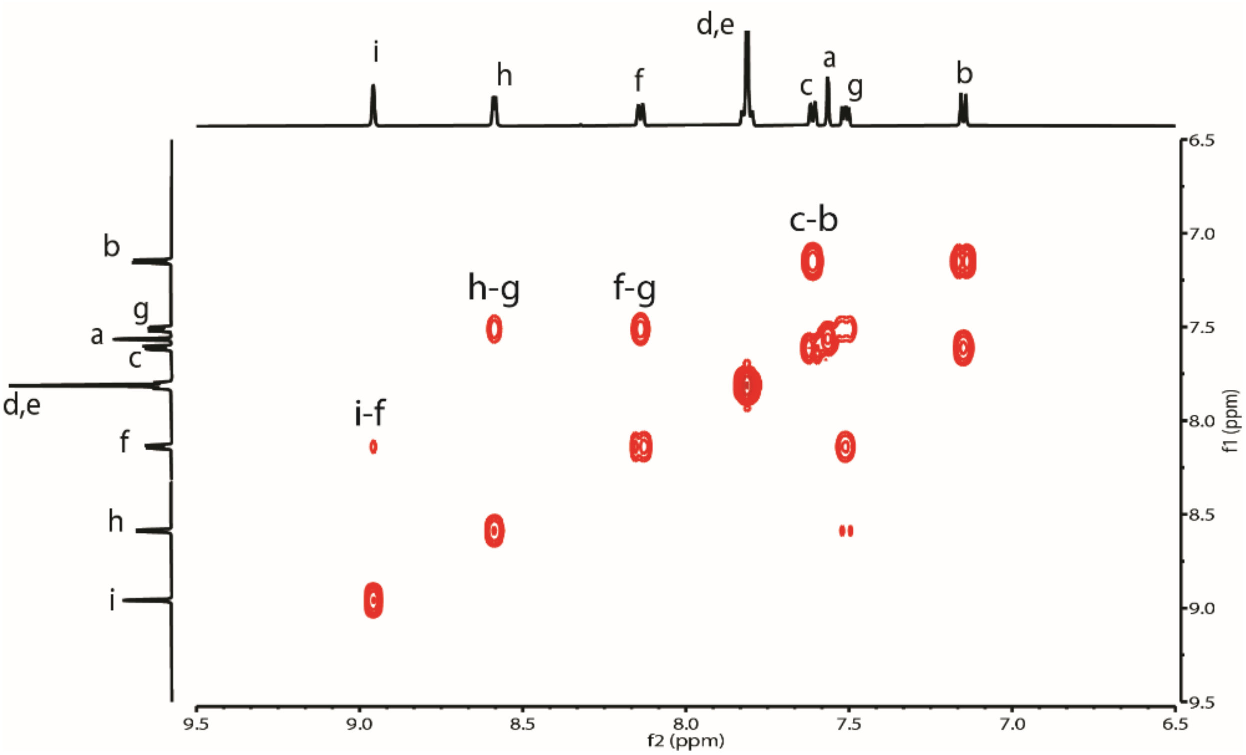

Figure S4: H – H COSY NMR spectrum (500 MHz, 298K, DMSO-d_6) of **L**.

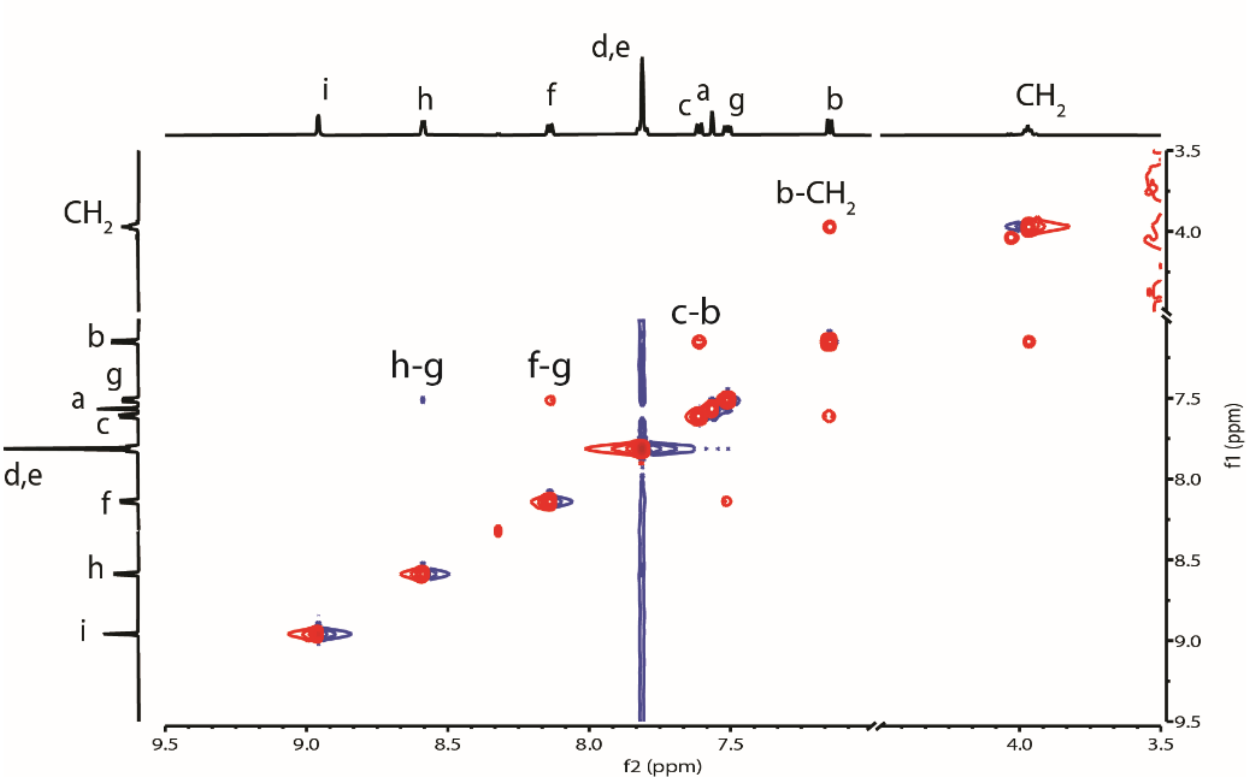

Figure S5: H – H NOESY NMR spectrum (500 MHz, 298K, DMSO-d_6) of **L**.

2.2 Cage 1

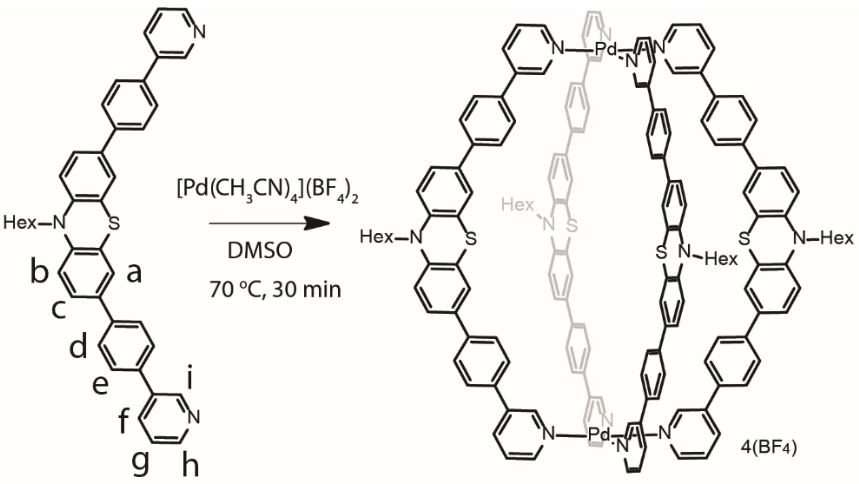

Scheme S2. Synthesis of cage **1**.

Cage **1** was formed in quantitative yield by heating a mixture of the ligand **L** (0.82 mg, 1.4 μmol, 2 equiv.) in DMSO-d_6 and a solution of $[Pd(CH_3CN)_4](BF_4)_2$ (0.75 μmol, 75 μL of a 10 mM solution in DMSO-d_6, 1 equiv.) at 70 °C for 30 min to give a solution of **1**.

^{1}H NMR (500 MHz, DMSO-d_6): δ [ppm] = 9.74 (d, J = 1.8 Hz, 2 H), 9.34 (dd, J = 5.2 Hz, 2 H), 8.44 (d, J = 8.4 Hz, 2 H), 7.86 (dd, J = 7.9 Hz, 2 H), 7.79-7.75 (m, 8 H), 7.53 (d, J = 6.5 Hz, 2 H), 7.49 (d, J = 2.0 Hz, 2 H), 7.07 (d, J = 8.5 Hz, 2 H), 3.96 (b, 2 H),1.76 (m, 2 H), 1.44 (m, 2 H), 1.30-1.25 (m, 4 H), 0.84 (t, J = 7.0 Hz, 3 H).

^{13}C NMR (126 MHz, 298 K, DMSO-d_6) δ [ppm] = 149.42, 148.74, 144.27, 139.83, 138.20, 132.91, 132.83, 127.68, 127.40, 126.73, 126.00, 124.89, 123.58, 116.22, 46.63, 30.84, 26.62, 25.87, 22.08, 13.82

ESI-FTICR-HRMS $[(C_{40}H_{35}N_3S)_4Pd_2]^{4+}$: found: 642.7095; calc.: 642.7078

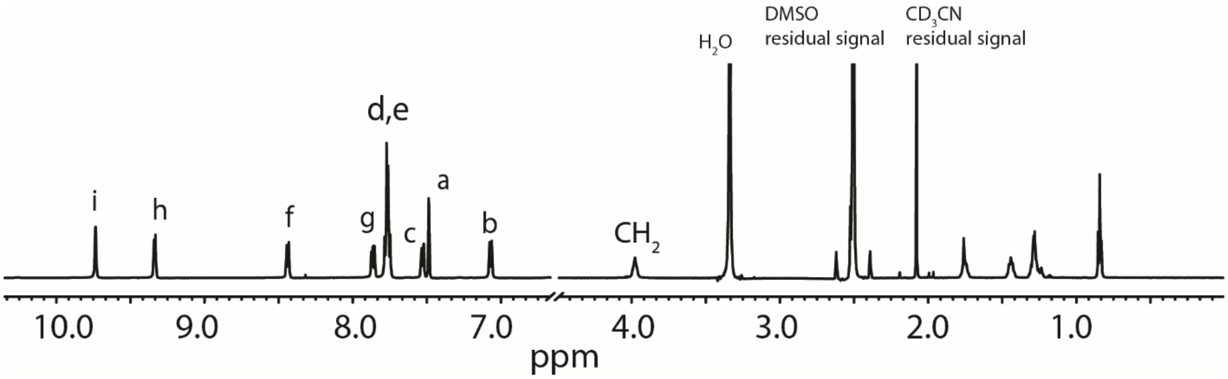

Figure S6: ^{1}H NMR spectrum (500 MHz, 298K, DMSO-d_6) of cage **1** (0.7 mM).

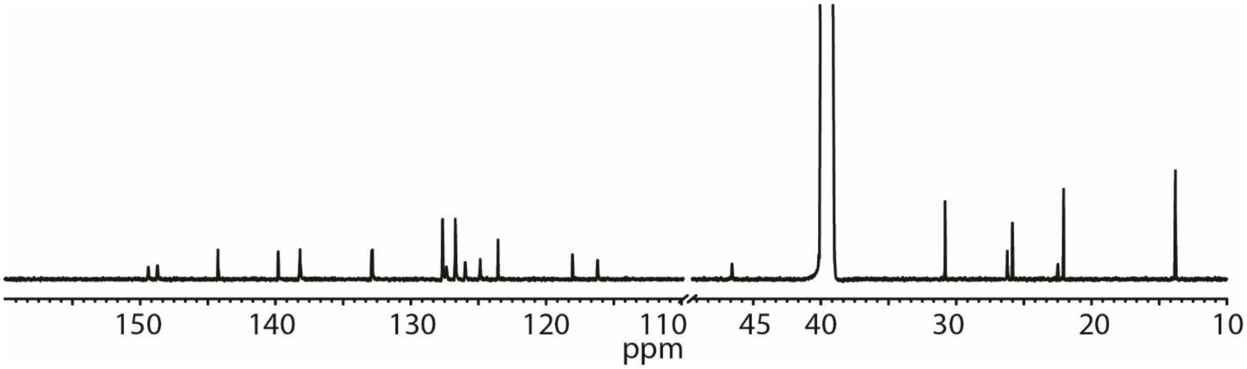

Figure S7: ^{13}C NMR spectrum (125 MHz, 298K, DMSO-d_6) of cage **1** (0.7 mM).

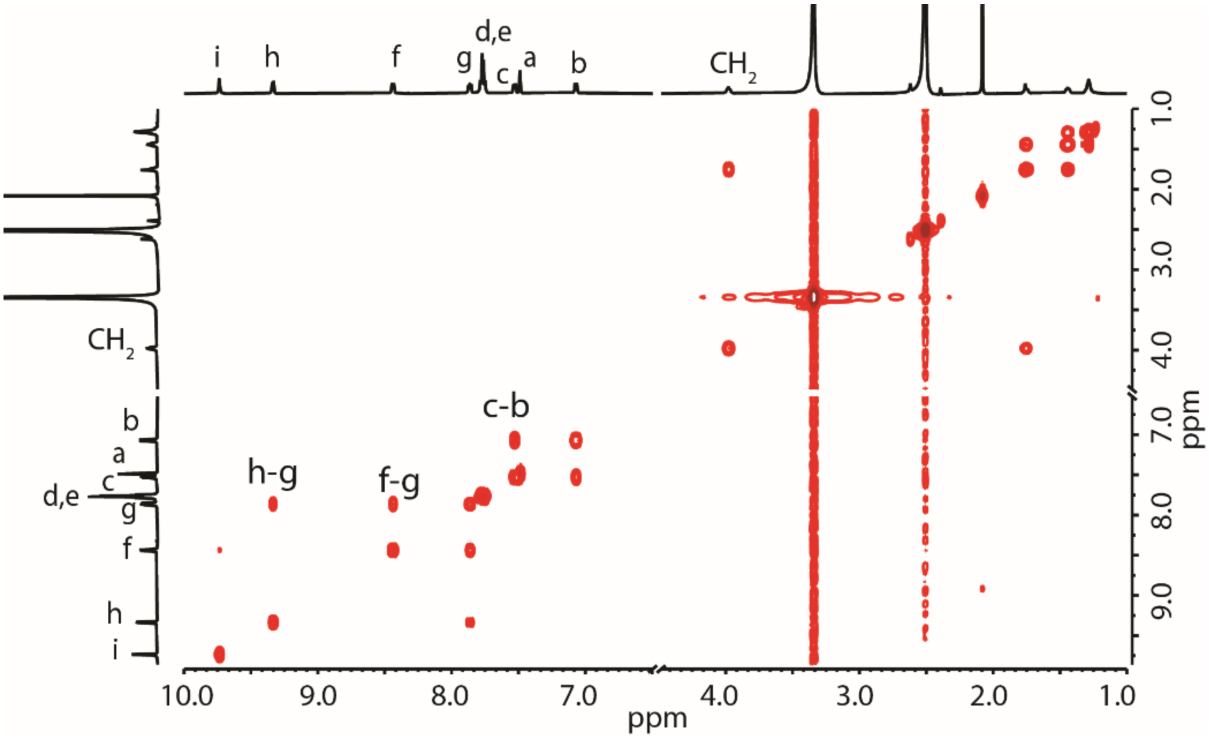

Figure S8: H – H COSY NMR spectrum (500 MHz, 298K, DMSO-d_6) of cage **1**

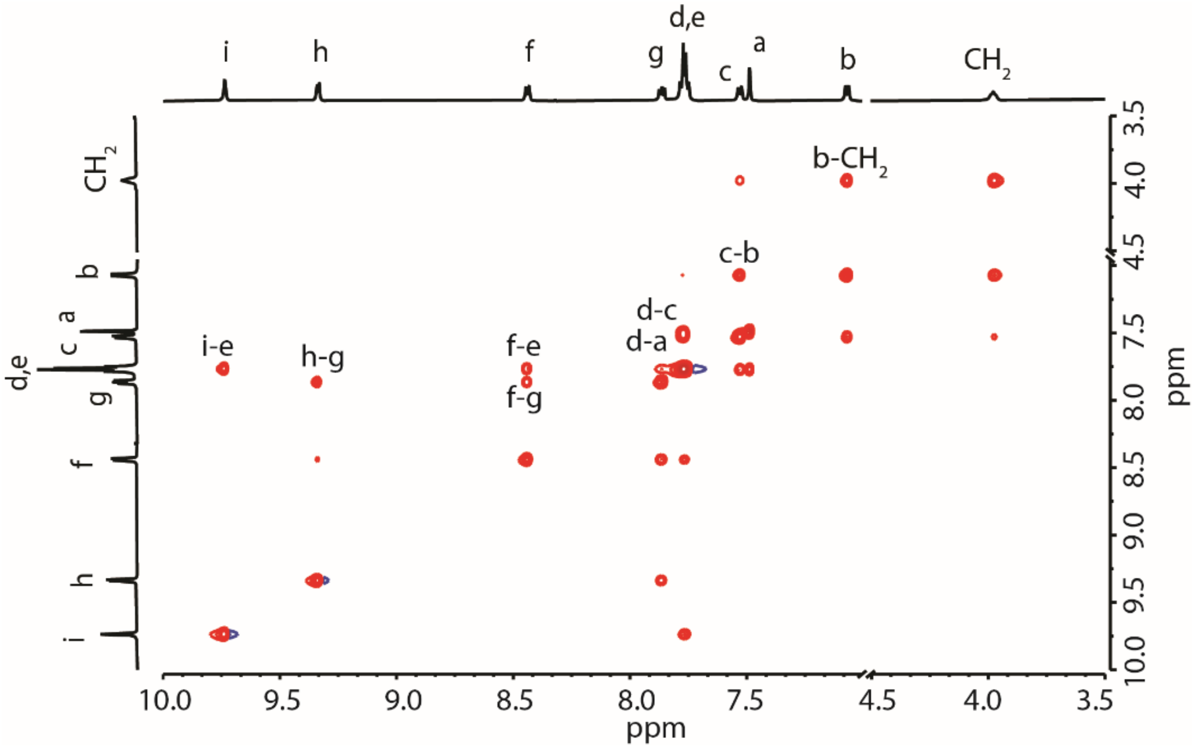

Figure S9: H-H NOESY NMR spectrum (500 MHz, 298K, DMSO-d_6) of cage **1**.

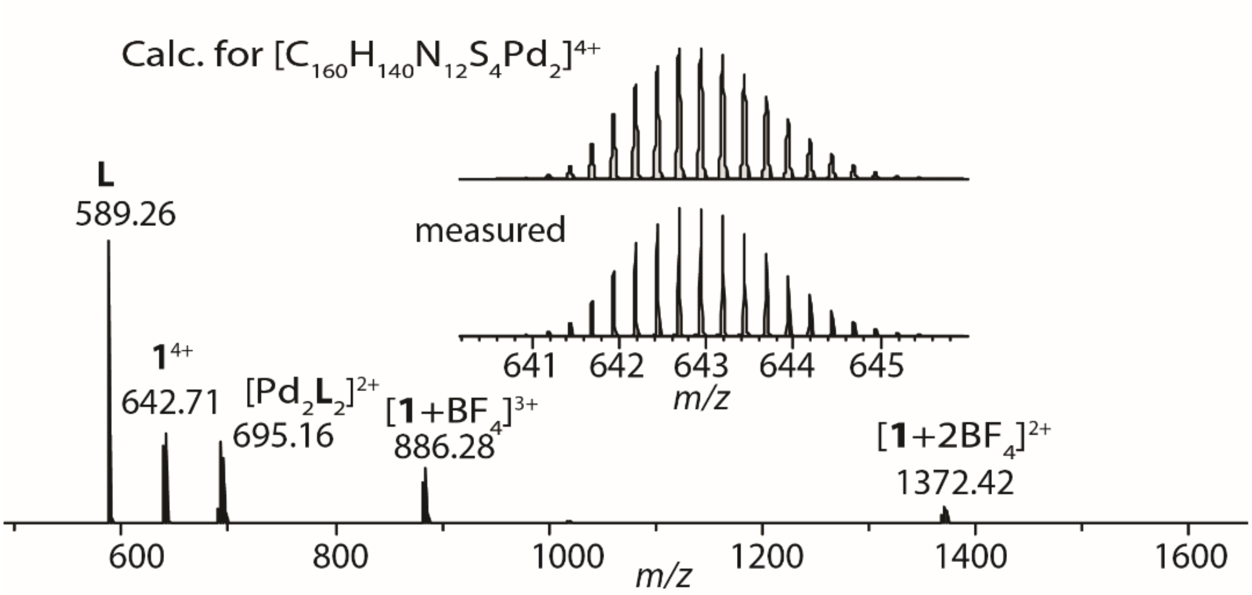

Figure S10: ESI-MS of cage **1**.

2.3 Host-guest supramolecular donor-acceptor systems:

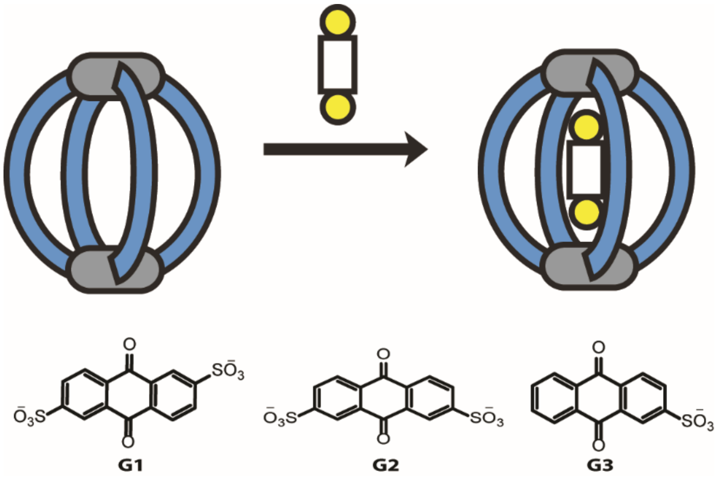

Figure S11: Schematic representation of guest encapsulation.

[**G1@1**]$^{2+}$ was formed in quantitative yield by adding **G1** (0.35 µmol, 35 µL of 10 mm in DMSO-d_6, 1 equiv.) to a solution of cage **1** (0.35 µmol, 500 µL of a 0.70 mM solution in DMSO-d_6, 1 equiv.) at room temperature for 30 min to give a solution of **G1@1**.
Although **G2** was added in similar way, only peak broadening without any change in the peak position was observed. This is might be due to larger agglomerates formed by the positively charged cage and negatively charged sulfonate anion **G2** that is obviously too large to fit inside the cavity.
[**G3@1**]$^{3+}$ was formed by adding **G3** (0.35 µmol, 35 µL of 10 mm in DMSO-d_6, 1 equiv.) to a solution of cage **1** (0.35 µmol, 500 µL of a 0.70 mM solution in DMSO-d_6, 1 equiv.) at room temperature for 30 min to give a solution of **G3@1**. The broadening of signal for inward pointing proton (H) indicates fast exchange of guest with the cage at 0.7 mM.

2.3.1 **$[G1@1]^{2+}$:**

H NMR (500 MHz, DMSO- d_6): δ [ppm] = 10.38 (b, 4 H), 9.43 (d, J = 5.4 Hz, 4 H), 8.94 (s, 1 H), 8.66 – 8.62 (m, 1 H), 8.45 (d, J = 7.5 Hz, 4 H), 8.06 (d, J = 5.6 Hz, 8 H), 7.84 (t, J = 7.5 Hz, 4 H), 7.66 (d, J = 7.5 Hz, 8H), 7.44 (b, 8 H), 6.99 (d, J = 7.8 Hz, 4 H), 3.87 (b, 4 H),1.67 (b, 4 H), 1.36 (m, 4 H), 1.23 (b, 8 H), 0.79 (t, J = 7.0 Hz, 6 H).

ESI-FTICR-HRMS $[(C_{40}H_{35}N_3S)_4Pd_2(C_{14}H_6O_8S_2)]^{2+}$: found: 1468.8886; calc.: 1468.8917

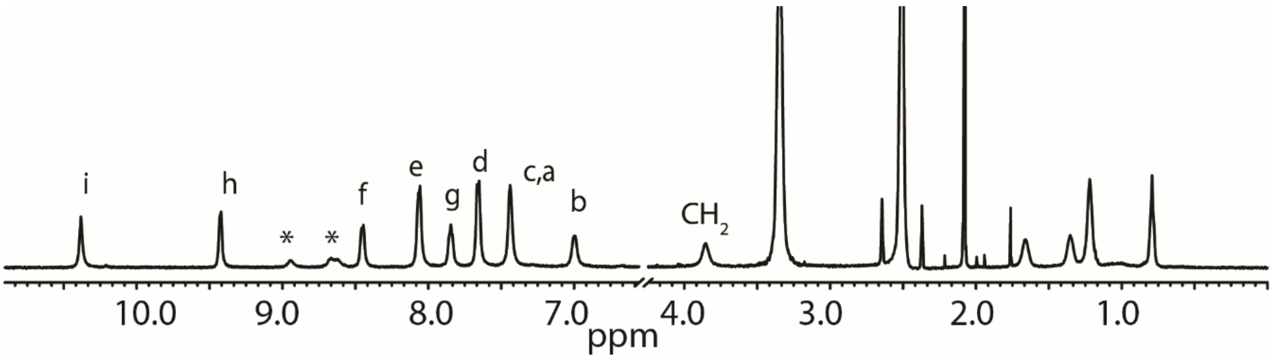

Figure S12: ^{1}H NMR spectrum (500 MHz, 298K, DMSO-d_6) of [**G1@1**]$^{2+}$ (0.7 mM). (**G1** signals are indicated by *).

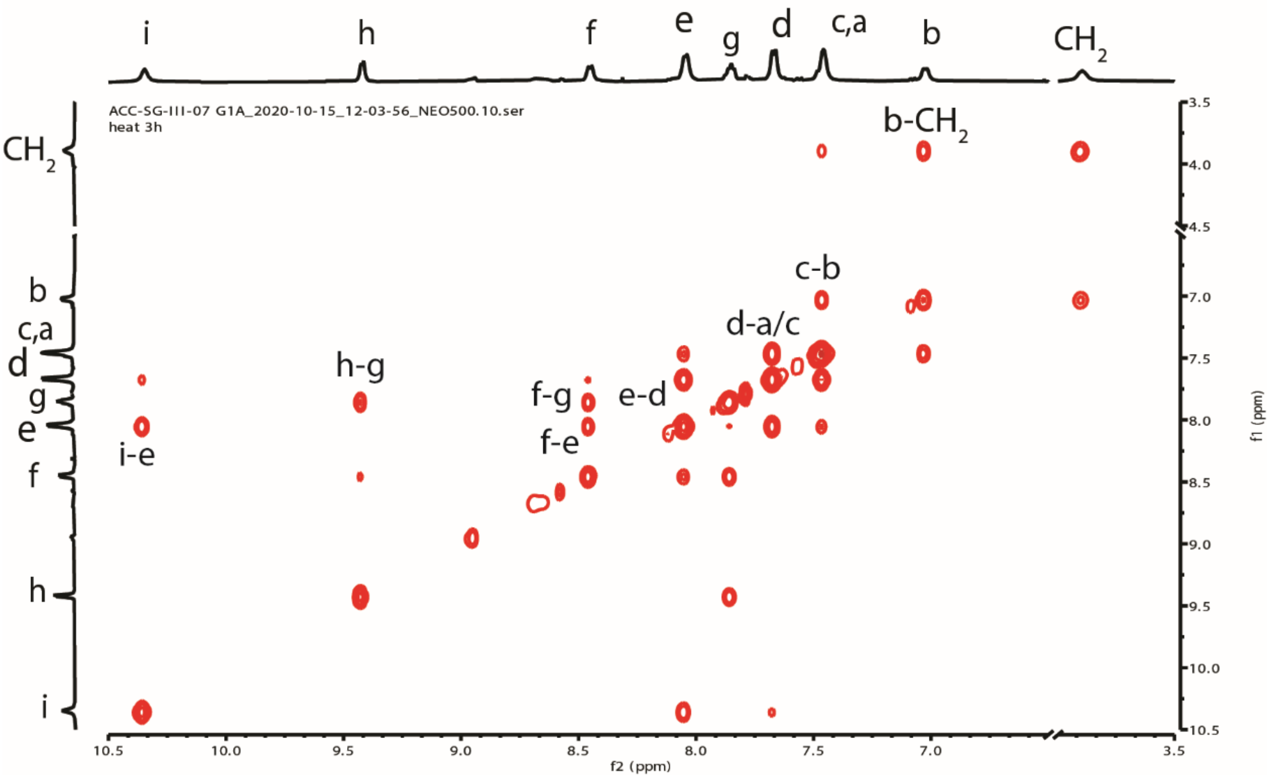

Figure S13: H-H NOESY NMR spectrum (500 MHz, 298K, DMSO-d_6) of [**G1@1**]$^{2+}$.

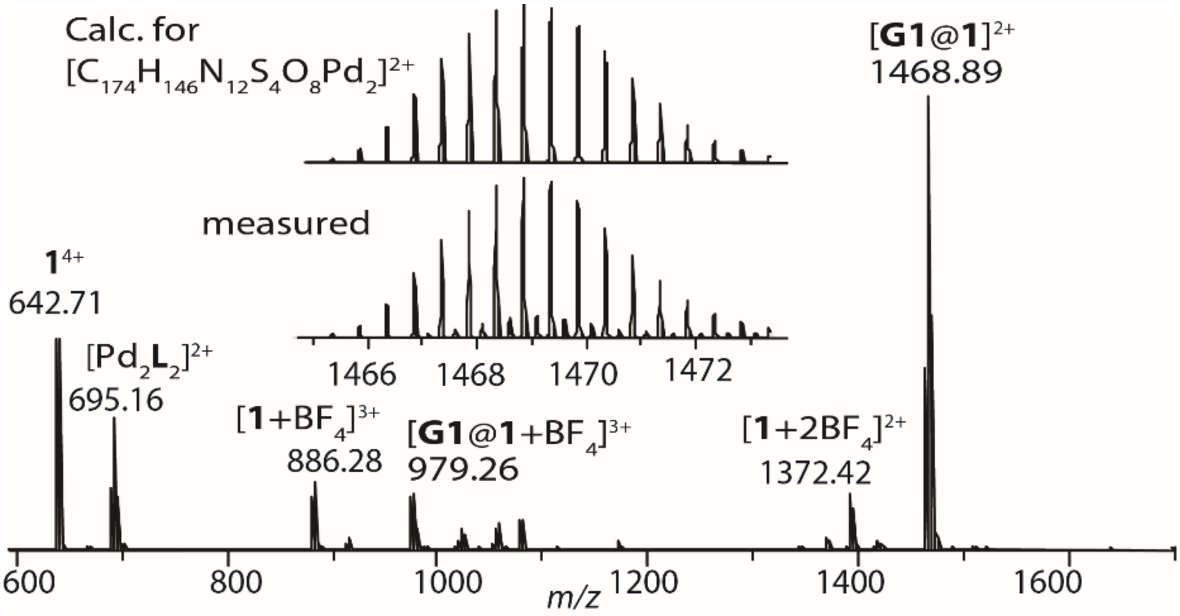

Figure S14: ESI-MS of [**G1@1**]$^{2+}$.

2.3.2 **G2@1:**

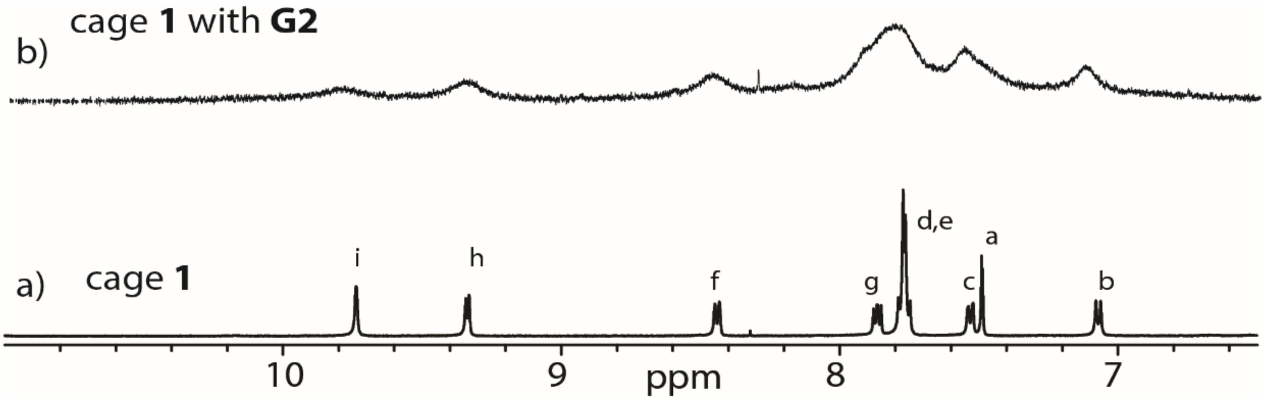

Figure S15: ^{1}H NMR spectrum (500 MHz, 298K, DMSO-d_6) of **G2** with cage **1** (0.7 mM).

2.3.3 **[G3@1]$^{3+}$:**

^{1}H NMR (500 MHz, DMSO-d_6): δ [ppm] = 9.84 (b, 8 H), 9.36 (d, J = 5.4 Hz, 8 H), 8.57 (b, 1 H), 8.44 (d, J = 7.9 Hz, 8 H), 8. 26-8.18 (m, 4 H), 7.87-7.75 (b, 40 H), 7.52 (d, J = 8.0 Hz, 8H), 7.44 (b, 8 H), 7.06 (d, J = 8.2 Hz, 8 H), 3.96 (b, 8 H),1.74 (b, 8 H), 1.43 (b, 8 H), 1. 28-1.23 (b, 16 H), 0.83 (t, J = 7.2 Hz, 12 H).

ESI-FTICR-HRMS $[(C_{40}H_{35}N_3S)_4Pd_2(C_{14}H_7O_5S)]^{3+}$: found: 952.9449; calc.: 952.9447

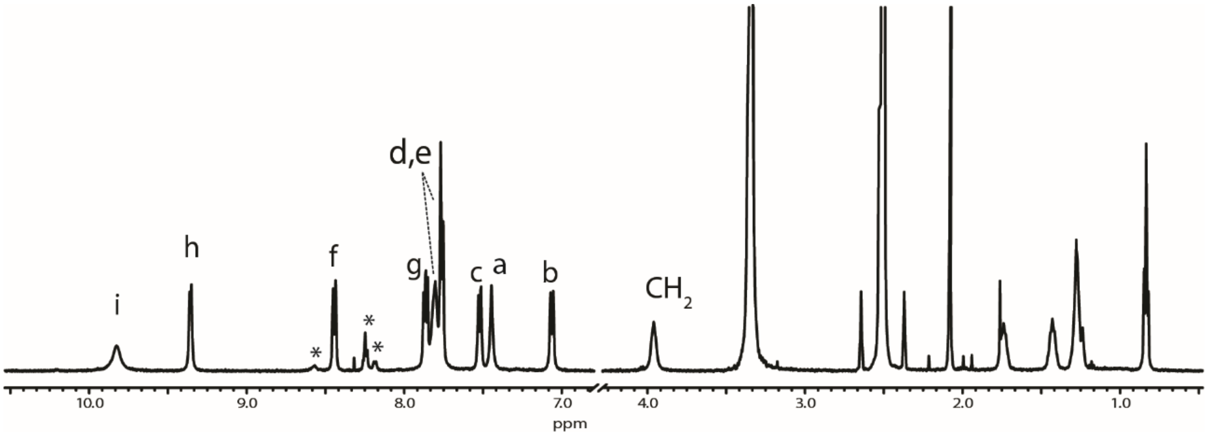

Figure S16: ^{1}H NMR spectrum (500 MHz, 298K, DMSO-d_6) of [**G3@1**]$^{3+}$ (0.7 mM). (**G3** signals are indicated by *)

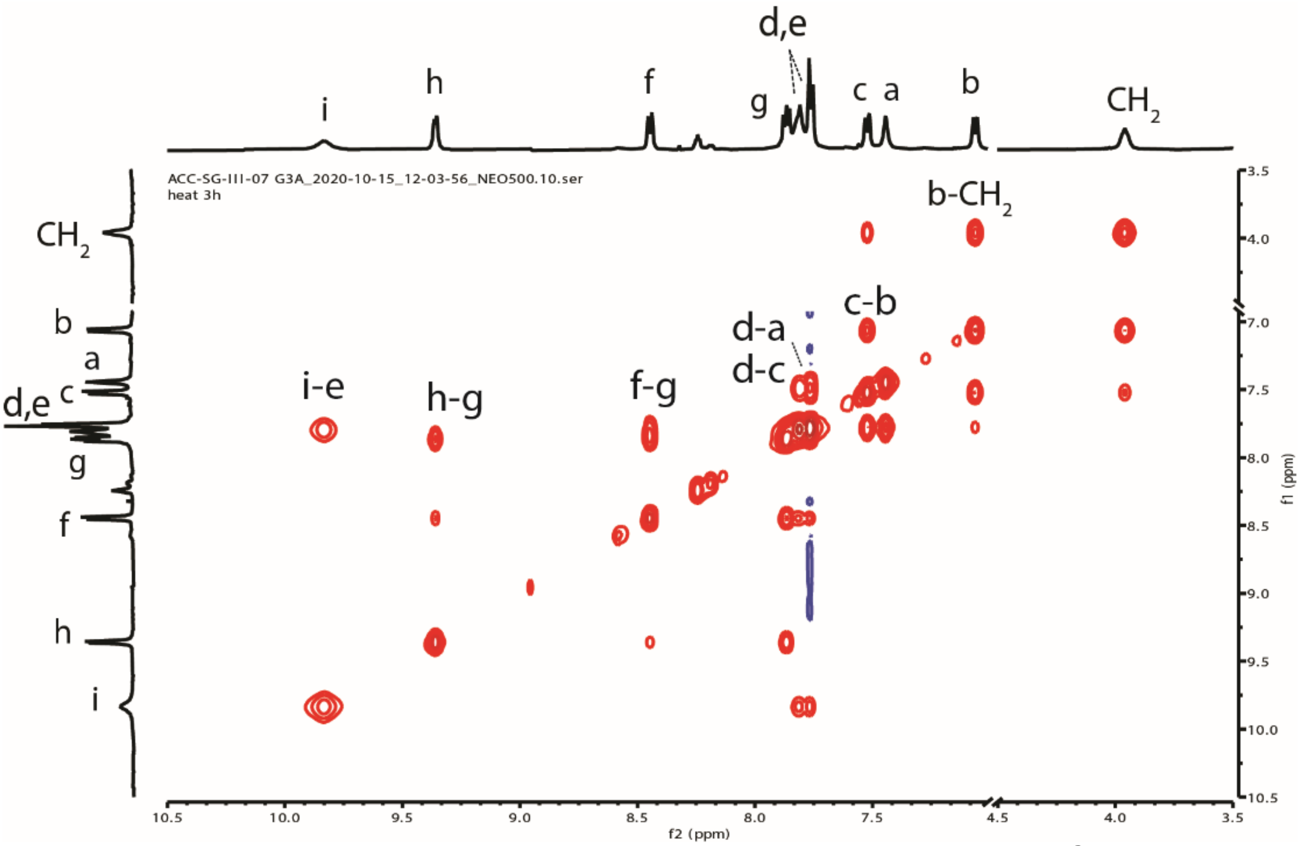

Figure S17: H-H NOESY NMR spectrum (500 MHz, 298K, DMSO-d_6) of [**G**3@**1**]$^{3+}$.

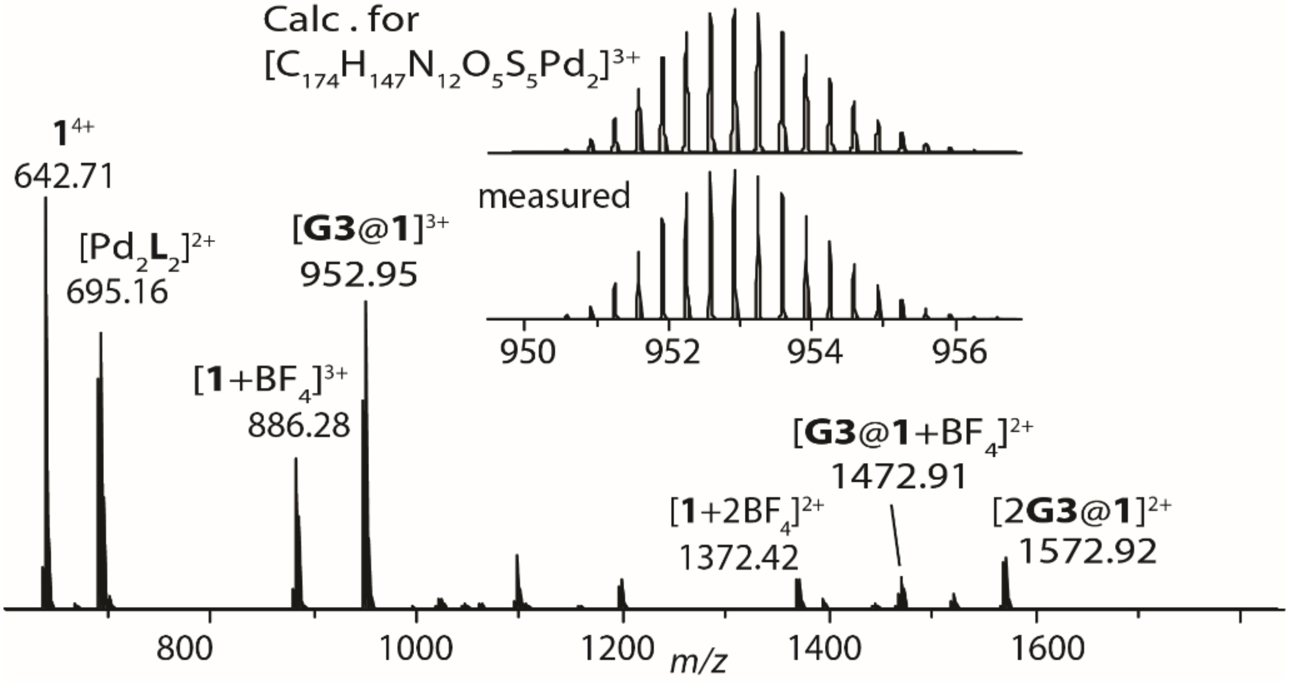

Figure S18: ESI-MS of [**G3**@**1**]$^{3+}$.

2.3.4 **^{1}H NMR titration experiments:**

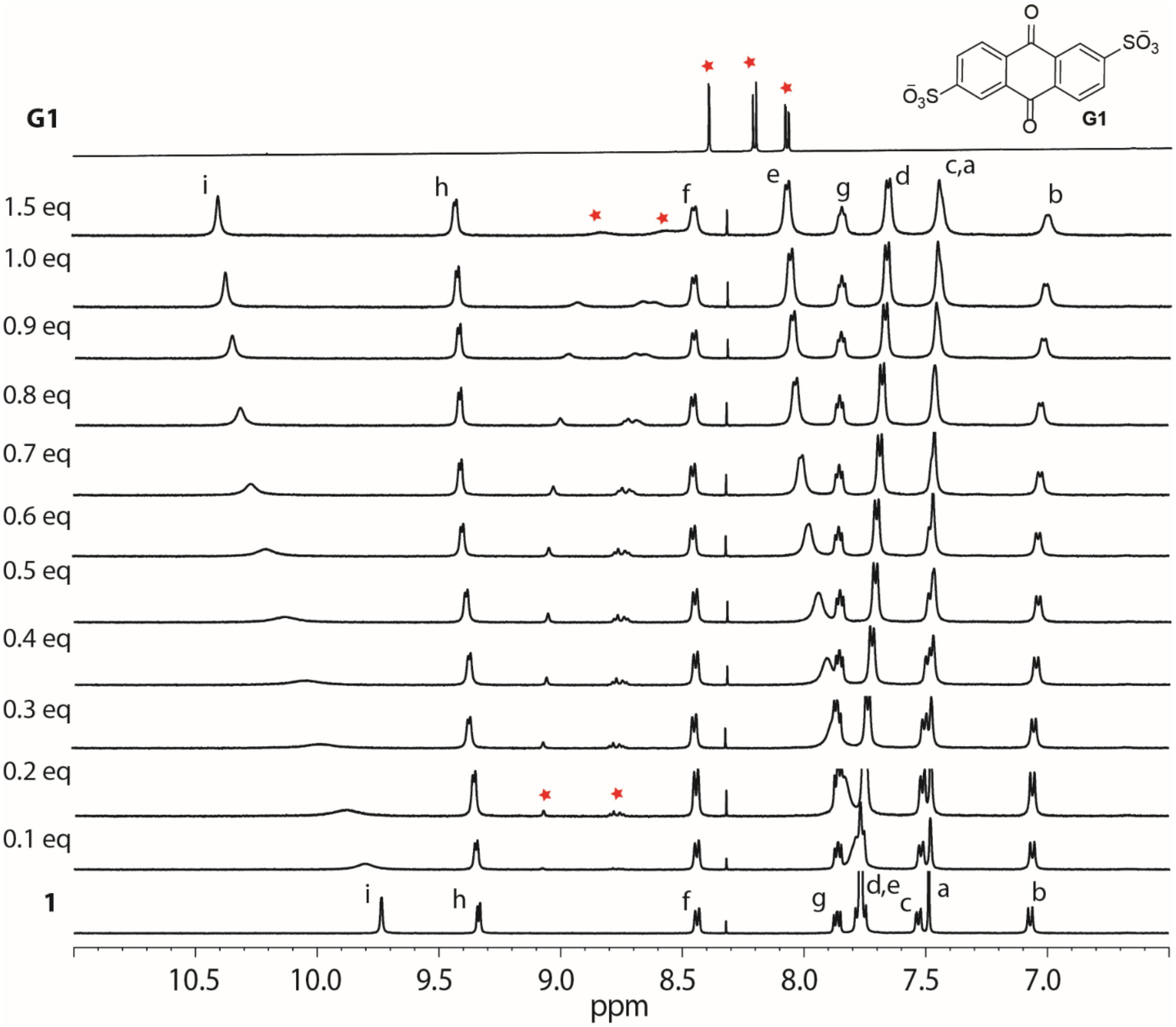

Figure S19: ^{1}H NMR spectrum (500 MHz, 298K, DMSO-d_6): Titration experiments of **G1** with cage **1**.

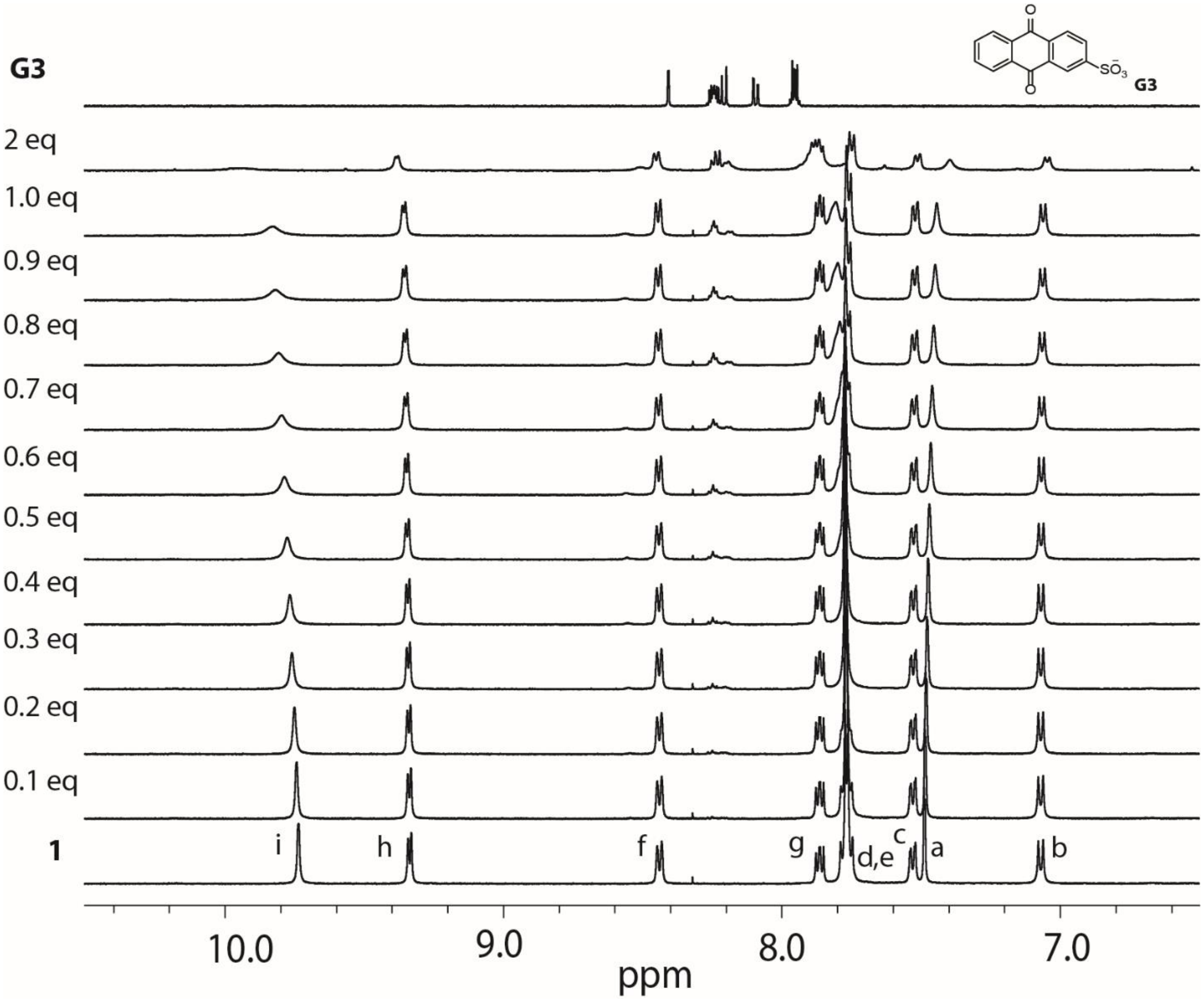

Figure S20: ^{1}H NMR spectrum (500 MHz, 298K, DMSO-d_6): Titration experiments of **G3** with cage **1**.

2.3.5 **Binding constant**

Binding constants were calculated form online bindfit software [4] using ^{1}H NMR titration experiments data.

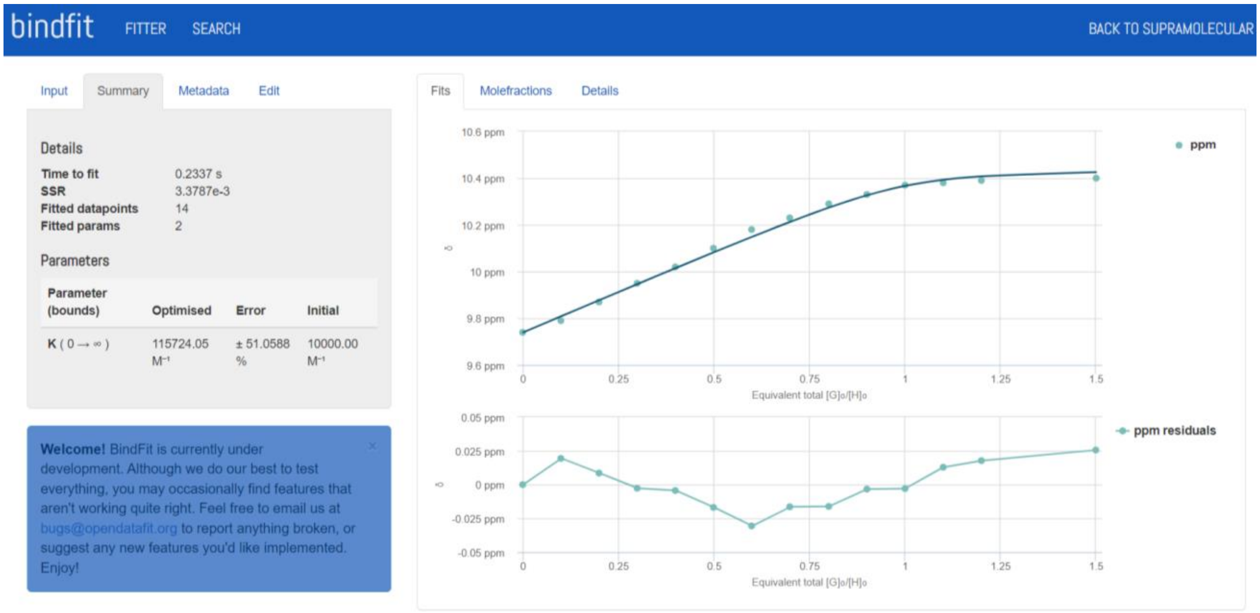

Figure S21: Binding constant calculation for **G1** (1.5 equiv.) with cage **1** (1.0 equiv., 0.7 mM). Further increase in the concentration of guest led to form agglomeration, followed by precipitation. From the bindfit software, the obtained binding constant K is ~1.16*10^{5} M^{-1} (± 60000).

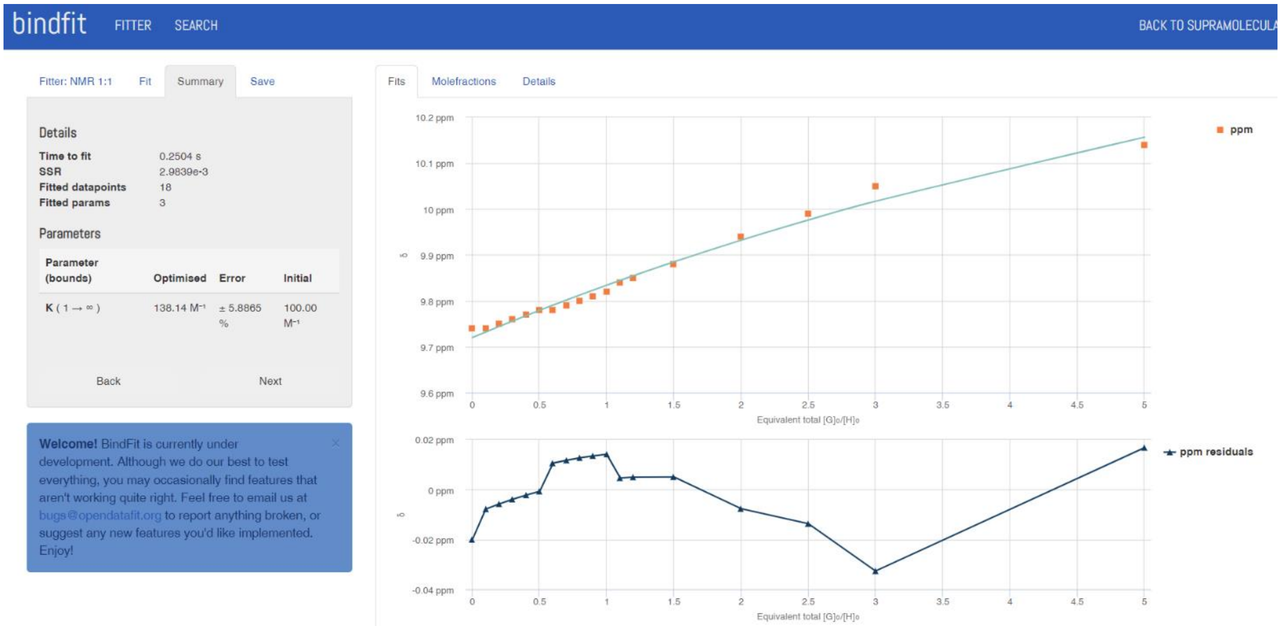

Figure S22: Binding constant calculation for **G3** (5.0 equiv.). with cage **1** (1.0 equiv., 0.7 mM). From the bindfit software, the obtained binding constant K is 138.14 M^{-1} (± 8).

2.3.6 **Guest selectivity by cage 1.**

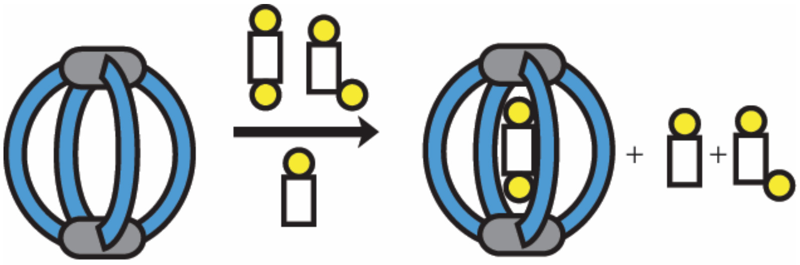

Figure S23: Schematic representation of guest selectivity of the cage **1**.

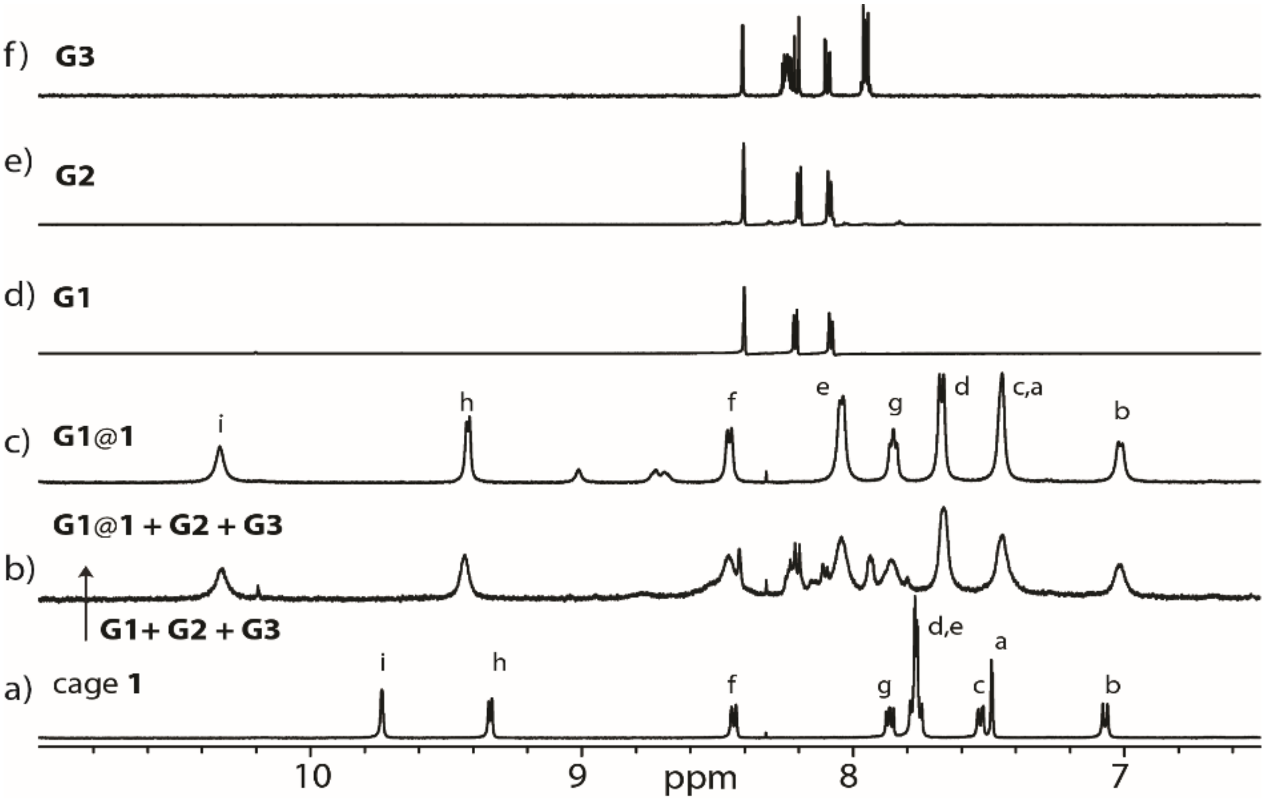

Figure S24: Guest selective ^{1}H NMR studies: ^{1}H NMR spectrum (500 MHz, 298K, DMSO-*d*$_6$) of a) cage **1**, b) 1 equiv. of each **G1**, **G2** and **G3** with cage **1**, c) [**G1@1**]$^{2+}$, d) **G1**, e) **G2**, and f) **G3**.

2.4 DOSY NMR studies:

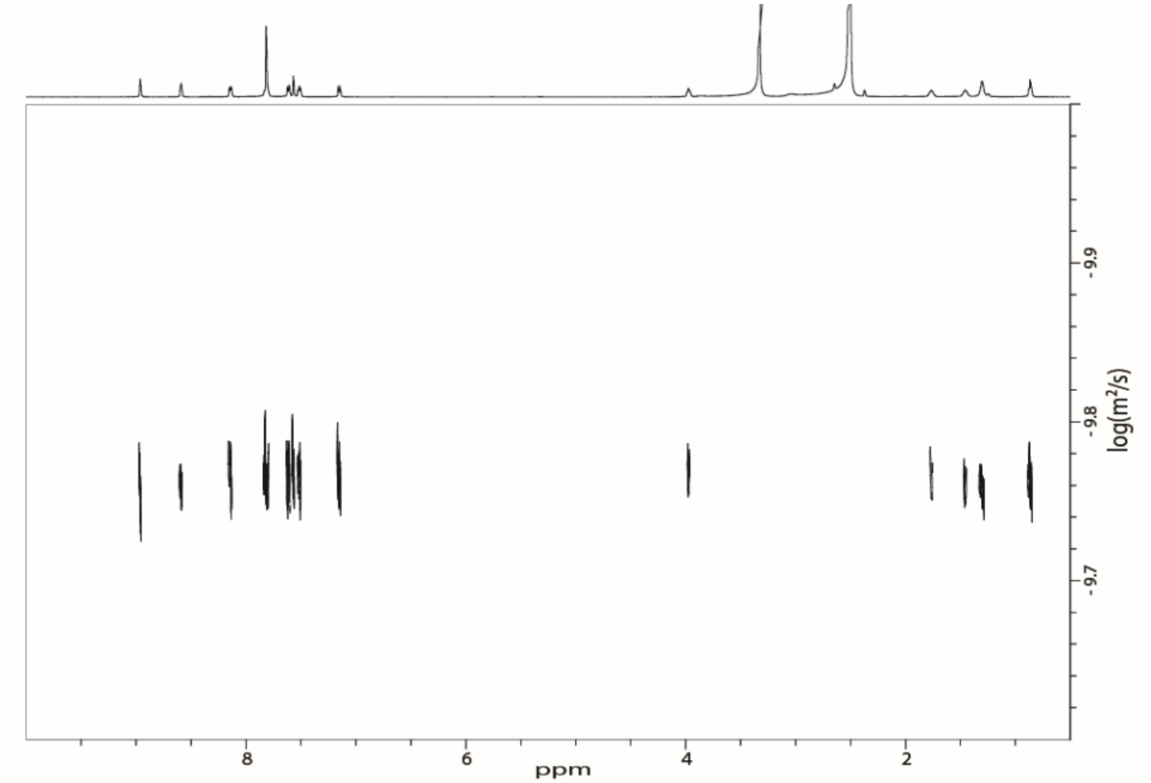

Figure S25: ^{1}H DOSY spectrum (500 MHz, 298K, DMSO-d_6) of ligand **L**.
Diffusion coefficient: 1.712×10^{-10} m^2s^{-1}, log D = -9.766. Hydrodynamic radius= 6.41 Å.

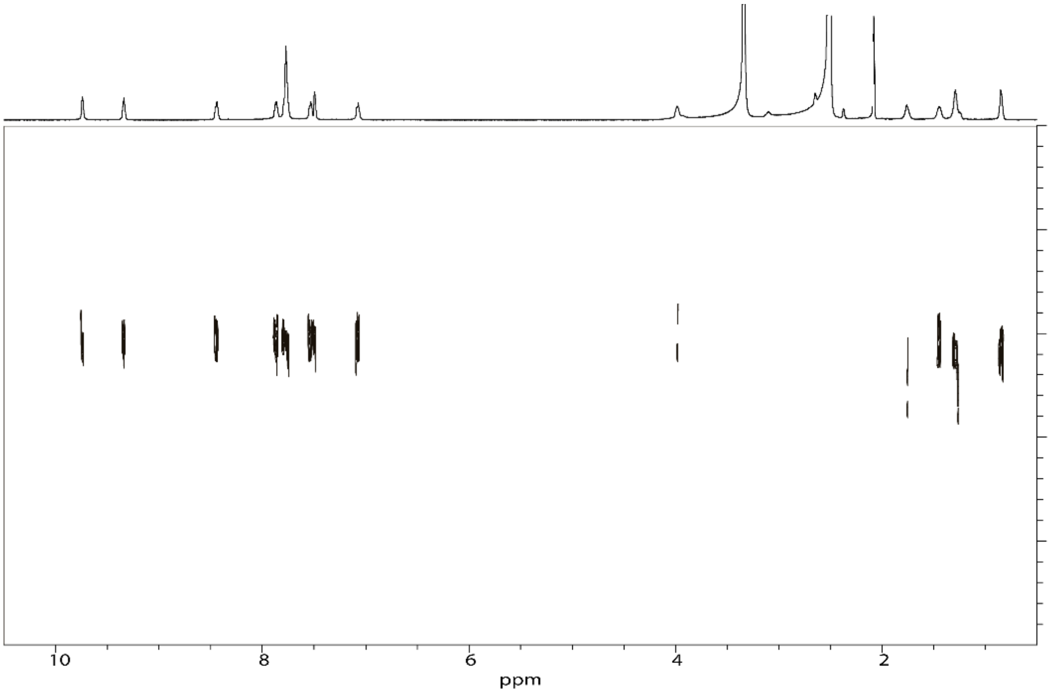

Figure S26: ^{1}H DOSY spectrum (500 MHz, 298K, DMSO-d_6) of cage **1**.
Diffusion coefficient: 8.049×10^{-11} m^2s^{-1}, log D = -10.094. Hydrodynamic radius= 13.63 Å.

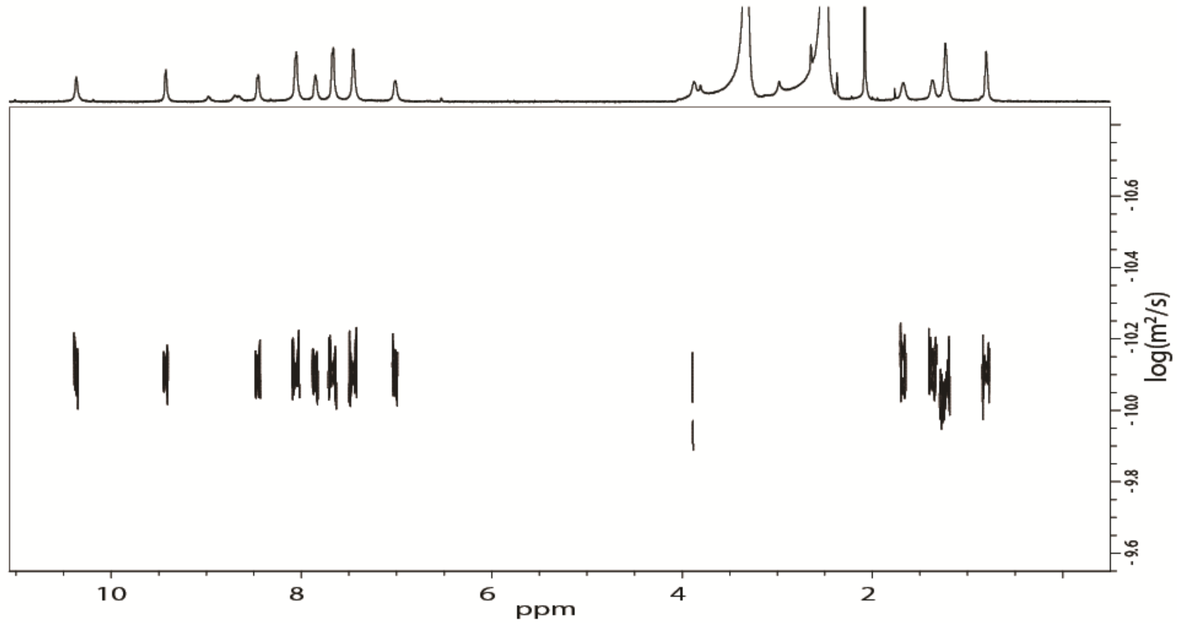

Figure S27: ^{1}H DOSY spectrum (500 MHz, 298K, DMSO-d_6) of [**G1**@**1**]$^{2+}$.
Diffusion coefficient: 7.873×10^{-11} m^2s^{-1}, log D = -10.094. Hydrodynamic radius= 13.93 Å.

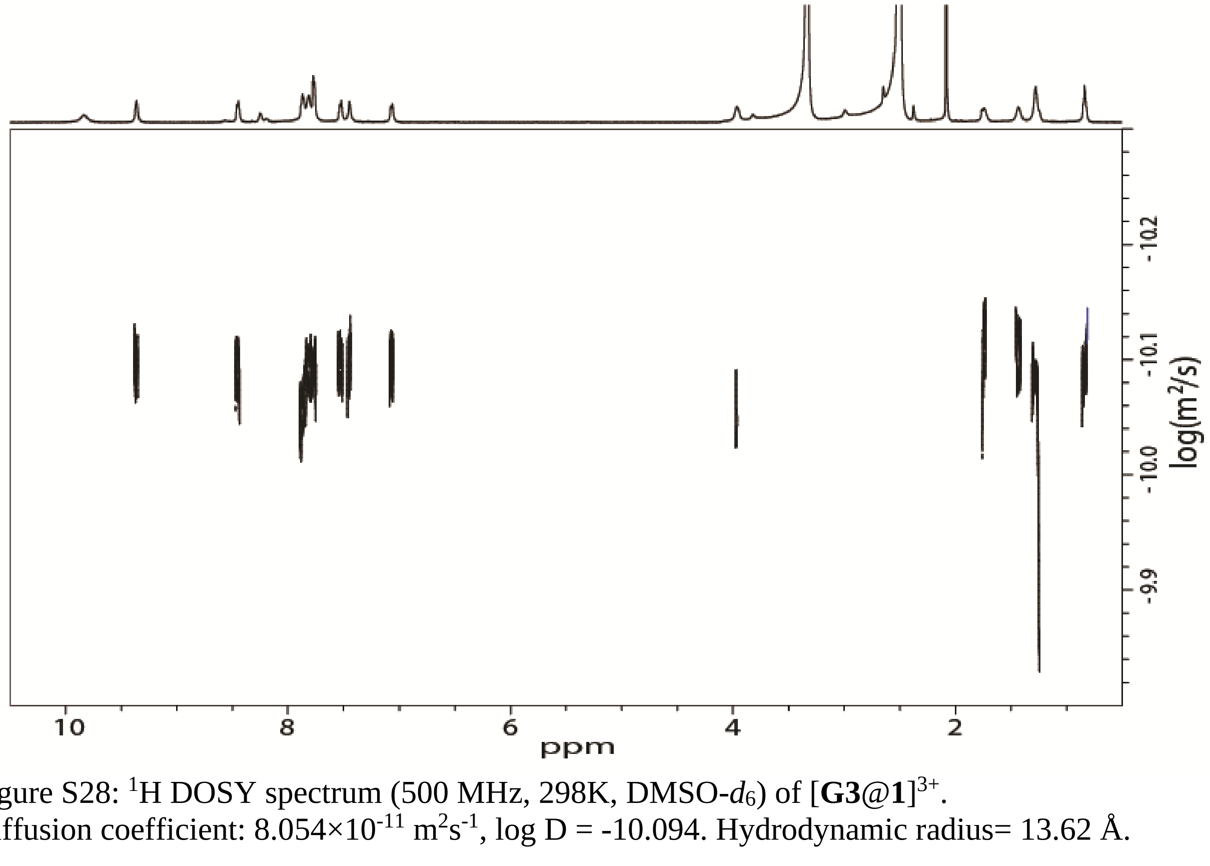

Figure S28: ^{1}H DOSY spectrum (500 MHz, 298K, DMSO-d_6) of [**G3**@**1**]$^{3+}$.
Diffusion coefficient: 8.054×10^{-11} m^2s^{-1}, log D = -10.094. Hydrodynamic radius= 13.62 Å.

3) Crystal structure

3.1 Table S1. Crystal data and structure refinement for ligand L, $[G1@1]^{2+}$

Compound	**L**	$[G1@1]^{2+}$
CIF ID	**sg6**	**sg88y_8**
CCDC number	**2114030**	**2114031**
Empirical formula	$C_{40}H_{35}N_3S$	$C_{401}H_{382}N_{24}O_{46}Pd_4S_{30}$
Formula weight	589.77	7660.68
Temperature [K]	100(2)	80(2)
Crystal system	monoclinic	triclinic
Space group (number)	$C2/c$ (15)	$P\bar{1}$ (2)
a [Å]	30.2629(9)	25.695(5)
b [Å]	7.1123(2)	29.288(6)
c [Å]	28.0446(8)	31.703(6)
α [Å]	90	99.01(3)
β [Å]	92.7290(10)	104.56(3)
γ [Å]	90	93.98(3)
Volume [Å^3]	6029.4(3)	22659(9)
Z	8	2
ρ_{calc} [g/cm^3]	1.299	1.123
μ [mm^{-1}]	1.208	0.322
F(000)	2496	7976
Crystal size [mm^3]	0.100×0.100×0.050	0.250×0.250×0.001
Crystal colour	yellow	yellow
Crystal shape	block	plate
Radiation	CuK_{α} (λ=1.54178 Å)	synchrotron (λ=0.6888 Å)
2Θ range [°]	6.31 to 133.15 (0.84 Å)	1.31 to 53.97 (0.76 Å)
Index ranges	-36 ≤ h ≤ 36<br>-8 ≤ k ≤ 8<br>-33 ≤ l ≤ 33	-33 ≤ h ≤ 33<br>-38 ≤ k ≤ 38<br>-41 ≤ l ≤ 40
Reflections collected	24489	333794
Independent reflections	5298<br>R_{int} = 0.0322<br>R_{sigma} = 0.0245	99649<br>R_{int} = 0.0543<br>R_{sigma} = 0.0525
Completeness to θ = 66.577°	99.4 %	93.3 %

Data / Restraints / Parameters	5298/0/398	99649/12500/4833
Goodness-of-fit on F^2	1.032	1.288
Final *R* indexes [$I \geq 2\sigma(I)$]	R_1 = 0.0313 wR_2 = 0.0837	R_1 = 0.1010 wR_2 = 0.3164
Final *R* indexes [all data]	R_1 = 0.0348 wR_2 = 0.0862	R_1 = 0.1275 wR_2 = 0.3427
Largest peak/hole [eÅ^3]	0.24/-0.28	3.29/-1.62

3.2 Crystal structure of L (sg6)

Yellow plate-shaped crystals of **L** (sg6) were grown by slow evaporation of a saturated solution of **L** in DMSO at room temperature. A single crystal in mother liquor was mounted onto a 0.1 mm nylon loop using NVH oil. Single crystal X-ray diffraction data was collected on a Bruker D8 venture equipped with an Incoatec microfocus source (Iμs 3.0) using Cukα radiation on a four axis κ-goniometer, equipped with an Oxford Cryostream 800 and a Photon II detector. All data was integrated with SAINT V8.40A and a multi-scan absorption correction using SADABS-2016/2 was applied. The space group was determined using XPREP.[5,6] The structure was solved by intrinsic phasing/direct methods using SHELXT[7] and refined with SHELXL[8] for full-matrix least-squares routines on F^2 and ShelXle[9] as a graphical user interface. All non-hydrogen atoms were refined with anisotropic displacement parameters. The hydrogen atoms were refined isotropically on calculated positions using a riding model with their U_{iso} values constrained to 1.5 times the U_{eq} of their pivot atoms for terminal sp3 carbon atoms and 1.2 times for all other carbon atoms.

Crystallographic data (including structure factors) for the structures reported in this paper have been deposited with the Cambridge Crystallographic Data Centre. CCDC **2114030** contains the supplementary crystallographic data for this structure. Copies of the data can be obtained free of charge from The Cambridge Crystallographic Data Centre via www.ccdc.cam.ac.uk/structures.

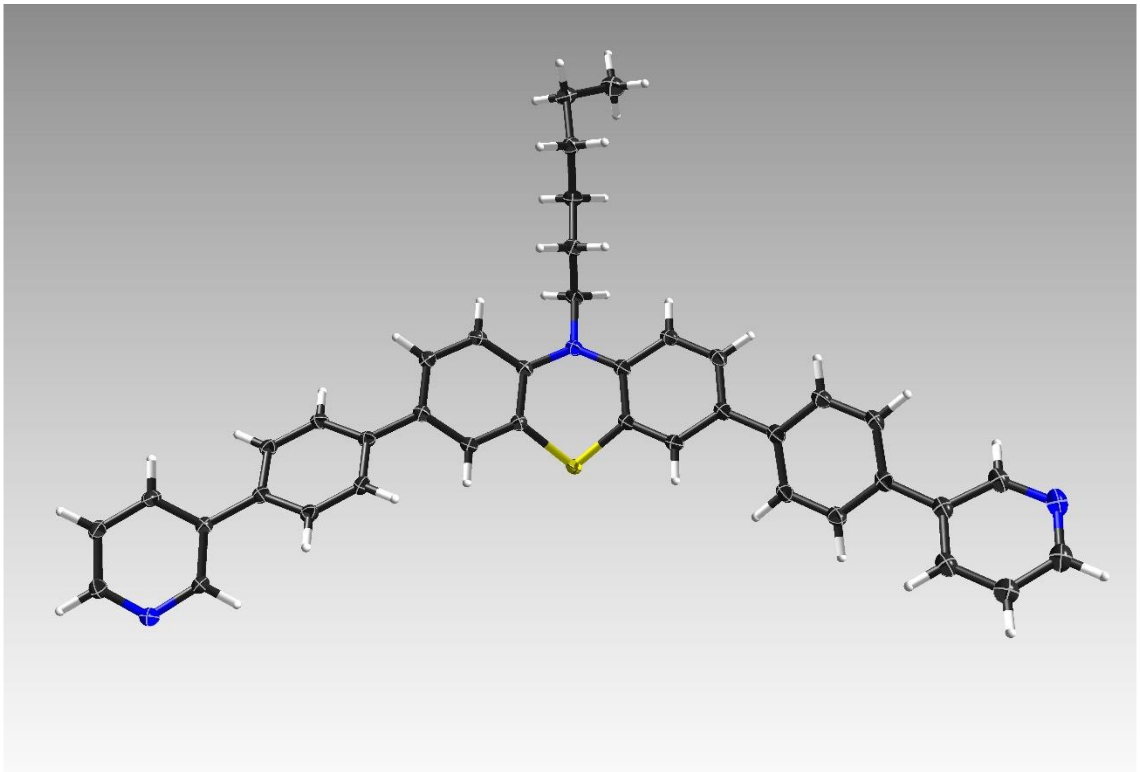

Figure 29. Crystal structure of ligand **L**. Anisotropic displacement parameters at 50% probability level.

3.3 Crystal structure of [G1@1]$^{2+}$ (sg88y_8)

Extremely thin plate-shaped single crystals of **[G1@1]$^{2+}$** were grown by slow diffusion of 1,4 dioxane into solution of **[G1@1]$^{2+}$** in DMSO over a period of four weeks. Single crystals of **[G1@1]$^{2+}$** in mother liquor was pipetted onto a glass slide containing NVH oil. To avoid cracking of the crystal, the crystal was quickly mounted onto a 200 µm nylon loop and immediately flash cooled in liquid nitrogen. Crystals were stored at cryogenic temperature in dry shippers, in which they were safely transported to macromolecular beamline P11 at Petra III,[5] DESY, Germany. A wavelength of λ = 0.6888 Å was chosen using a liquid N_2 cooled double crystal monochromator. Single crystal X-ray diffraction data was collected at 80(2) K on a single axis goniometer, equipped with an Oxford Cryostream 800 open flow cooling device and a Pilatus 6M fast detector. 3600 diffraction images were collected in a 360° φ sweep at a detector distance of 154 mm, 40,80% filter transmission, 0.1° step width and 0.1 seconds exposure time per image. Data integration and reduction were undertaken using XDS.[6] The structure was solved by intrinsic phasing/direct methods using SHELXT[7] and refined with SHELXL[8] using 22 cpu cores for full-matrix least-squares routines on F^2 and ShelXle[9] as a graphical user interface and the DSR program plugin was employed for modeling.[10]

The asymmetric unit contains two supramolecular coordination cages, four **G1** guest molecules as well as 14 DMSO solvents molecules. The subatomic resolution of 0.76 Å revealed disorder in four out of eight sulfonate groups of **G1** guest molecules (residue class ASO), three of the hexyl chains at ligand (residue class PPH) and as well as one of the DMSO solvents molecules (residue class DMS).

In each case, disorder was modelled with two discrete positions refining their occupancy factor using a free variable and ensuring sensible geometry by employing stereochemical restraints. Despite reaching subatomic resolution of 0.76 Å, disorder and poor crystal quality required stereochemical restraints to be employed for ensuring a sensible geometry of the organic part of the structure.

Stereochemical restraints for the ligands (residue class PPH), guest (residue class ASO) and DMSO solvents molecules (residue class DMS) were generated by the GRADE program using the GRADE Web Server (http://grade.globalphasing.org) and applied in the refinement. A GRADE dictionary for SHELXL contains target values and standard deviations for 1,2-distances (DFIX) and 1,3-distances (DANG), as well as restraints for planar groups (FLAT). All displacements for non-hydrogen atoms were refined anisotropically. The refinement of ADP's for carbon, nitrogen and oxygen atoms was enabled by a combination of similarity restraints (SIMU) and rigid bond restraints (RIGU).[11] The contribution of the electron density from disordered counterions and solvent molecules, which could not be modeled with discrete atomic positions were handled using the SQUEEZE[12] routine in PLATON.[13] The solvent mask file (.fab) computed by PLATON were included in the SHELXL refinement via the ABIN instruction leaving the measured intensities untouched.

CCDC **2114031** contain the supplementary crystallographic data for this structure. Copies of the data can be obtained free of charge from The Cambridge Crystallographic Data Centre via www.ccdc.cam.ac.uk/structures.

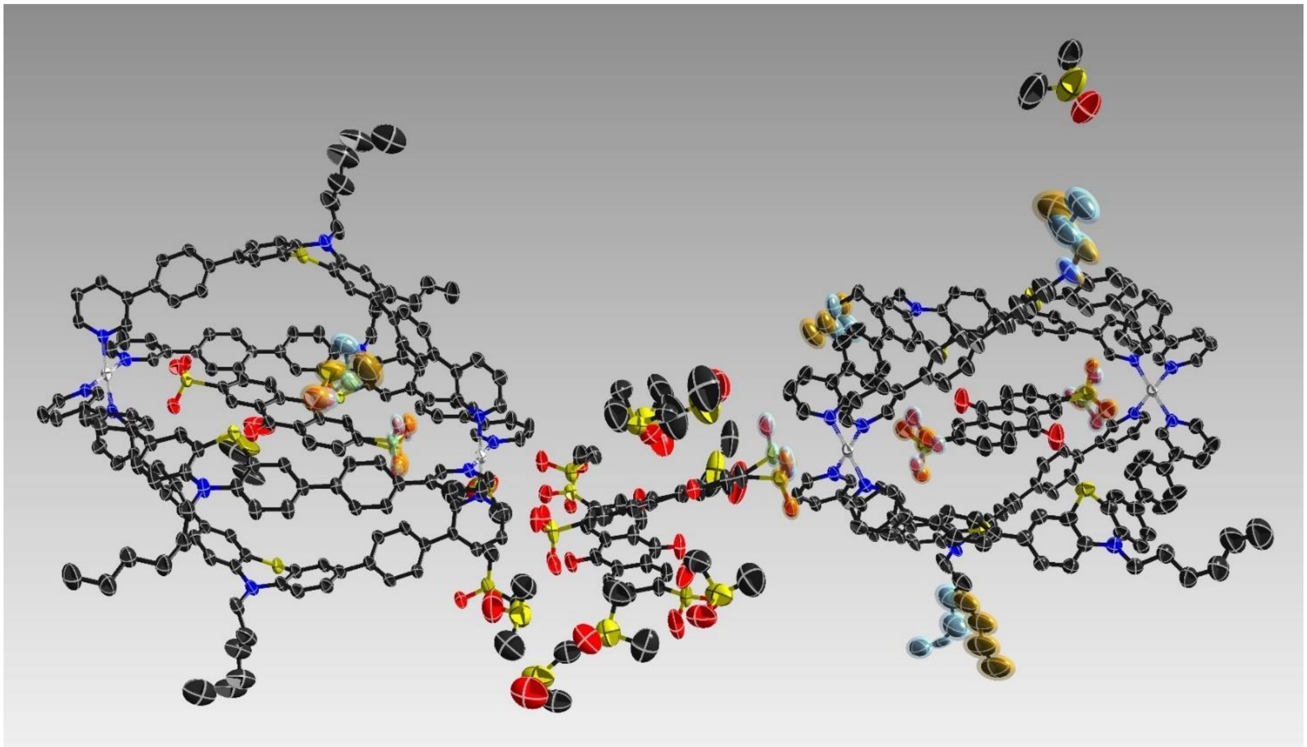

Figure 30: Asymmetric unit of host-guest assembly [**G1**@**1**]$^{2+}$: Asymmetric unit contains two molecules of [**G1**@**1**]$^{2+}$, two molecules of free guest **G1**, and fourteen DMSO molecules. Disorder highlighted in yellow/blue. Anisotropic displacement parameters (ADPs) at 50% probability level. Hydrogens omitted for clarity.

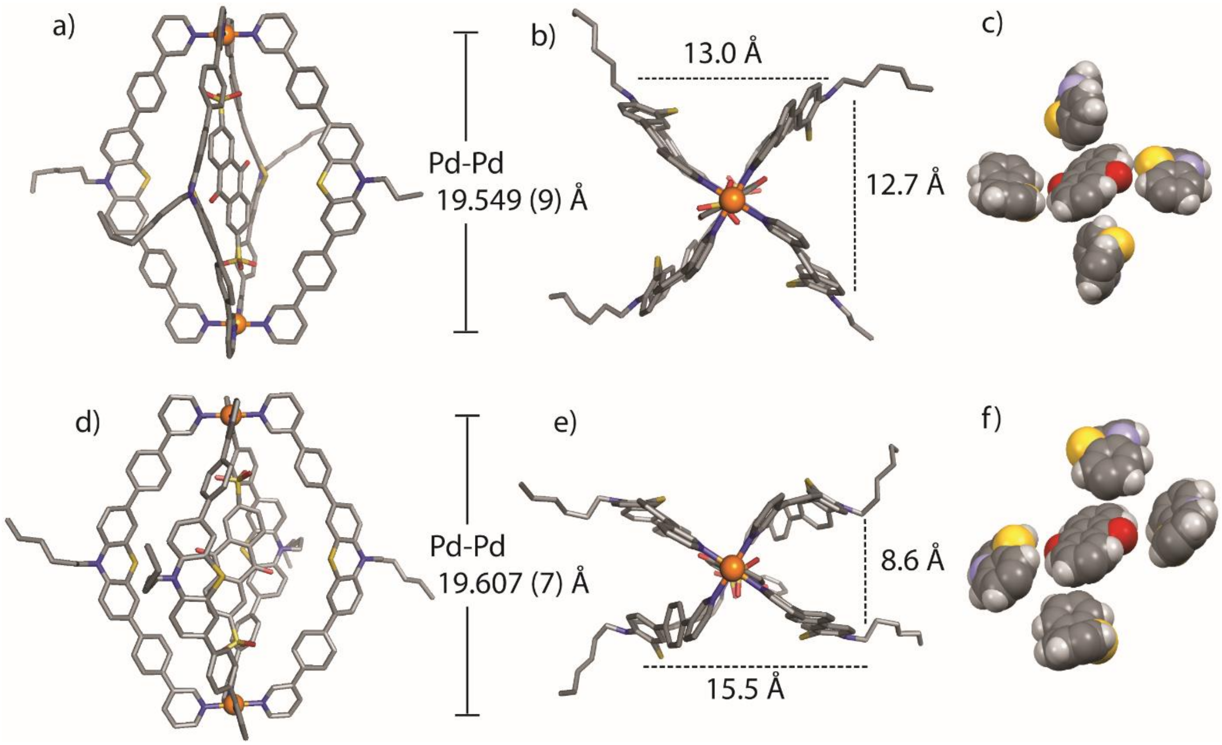

Figure 31: Two conformational isomers in the crystal structure of the host-guest assembly [**G1**@**1**]$^{2+}$: conformer A (**HG-A**) (a-c in the figure) and confirmer B (**HG-B**) (d-f in the figure), along Pd-Pd axis (a, d), via Pd-Pd axis (b, e), and spatial arrangement of the donor PTZ and acceptor AQ units (c, f) respectively. (Hydrogen atoms, solvent molecules and other **G1** anion molecules omitted for clarity).

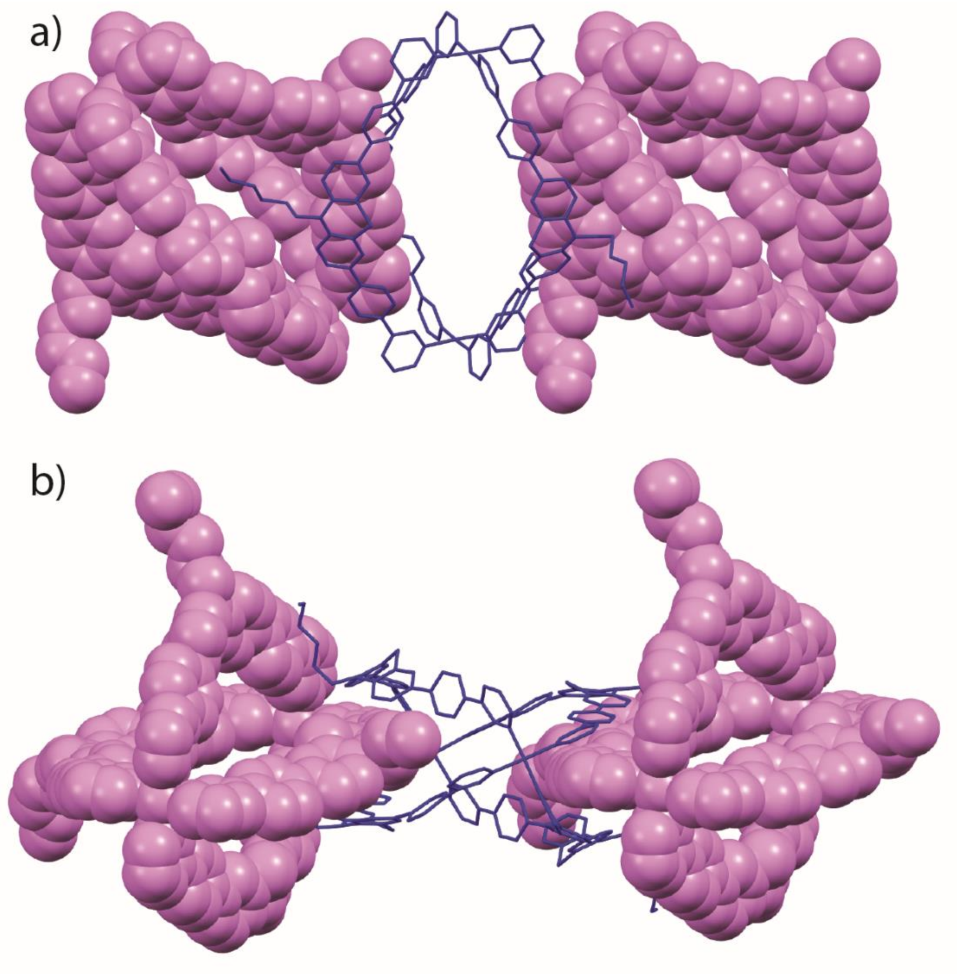

Figure 32: Close packing of two conformational isomers with the help of π - π stacking in the crystal structure of the host-guest assembly [**G1@1**]$^{2+}$: along Pd-Pd axis (a), via Pd-Pd axis (b). One of the ligands in conformer A (**HG-A**) (pink colour) is sandwiched between the two of the ligands of confirmer B (**HG-B**) (blue colour) with the help of π - π interaction. (Hydrogen, solvent, and other **G1** anion molecules omitted for clarity).

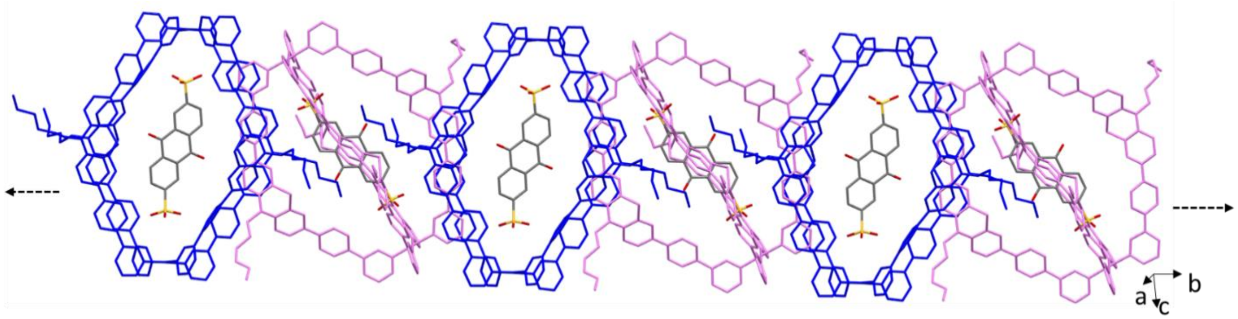

Figure 33: One dimensional π -network in the crystal structure of the host-guest assembly [**G1@1**]$^{2+}$.

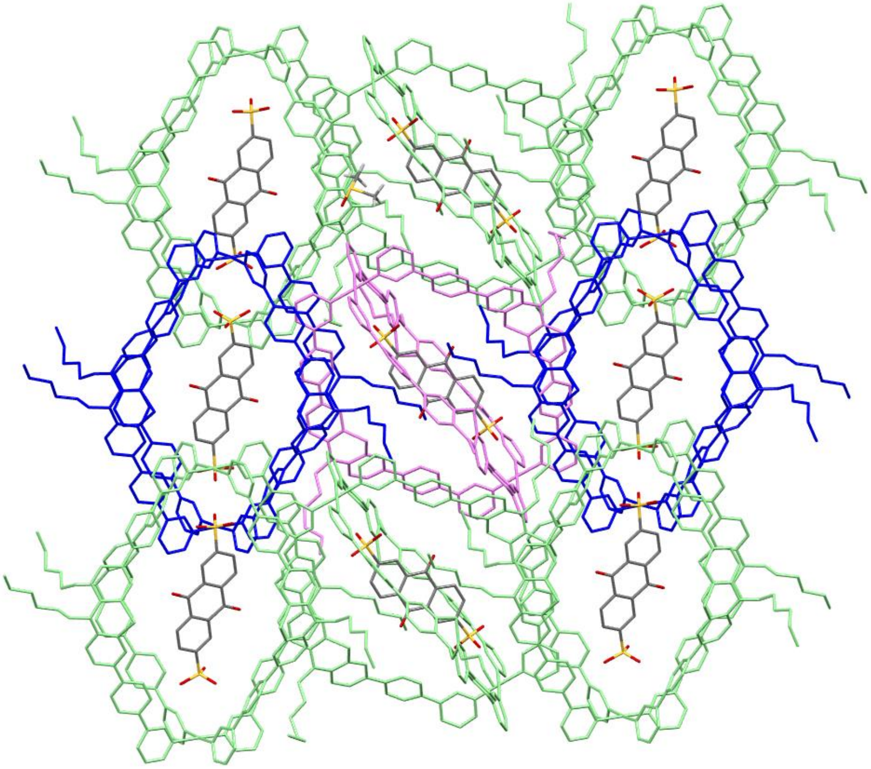

Figure 34: Two dimensional π -network in the crystal structure of the host-guest assembly [**G1**@**1**]$^{2+}$ (along axis a)

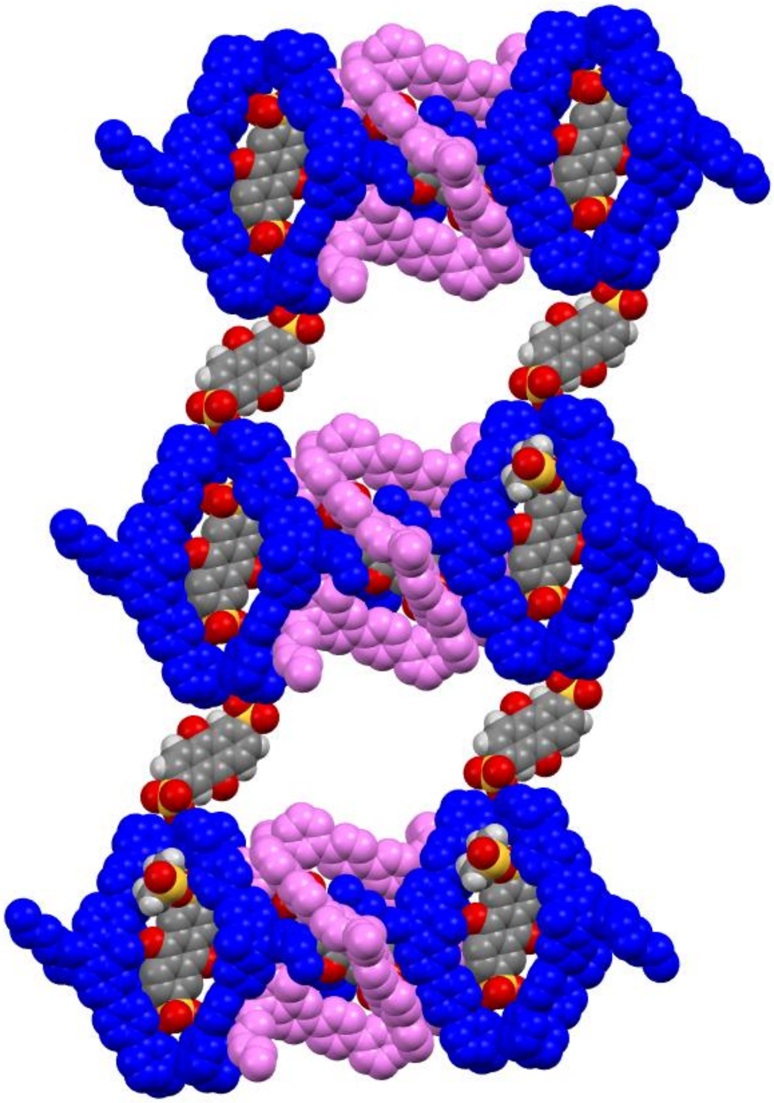

Figure 35: **G1** ions bridged between two of one dimensional π -network in the crystal structure of the host-guest assembly [**G1**@**1**] (along axis a).

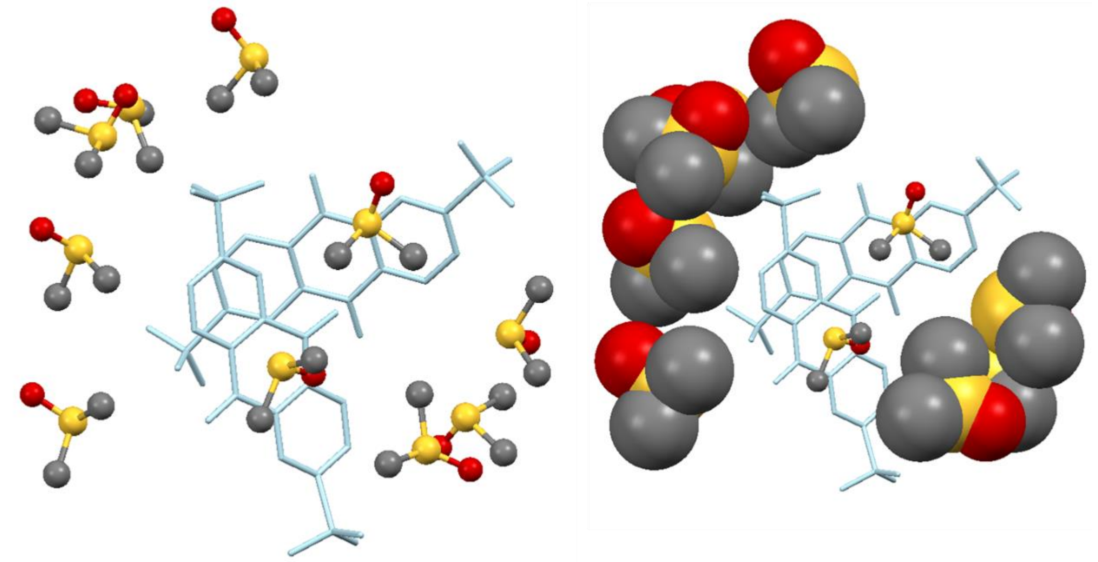

Figure 36: Arrangement of DMSO solvent molecules around **G1** ions (highlighted in blue) in the crystal structure of the host-guest assembly [**G1**@**1**]$^{2+}$.

4) Theoretical studies

4.1 Optimized structures

For the quantum mechanical calculations performed to obtain the following electronic structures, the ORCA software package, version 5.0.2, was used.[14] Geometry optimizations were done on B3LYP/def2-SVP level of theory without dispersion correction and implicit solvent. The final single point calculations were done on B3LYP/def2-TZVP level using the CPCM(DMSO) software model.

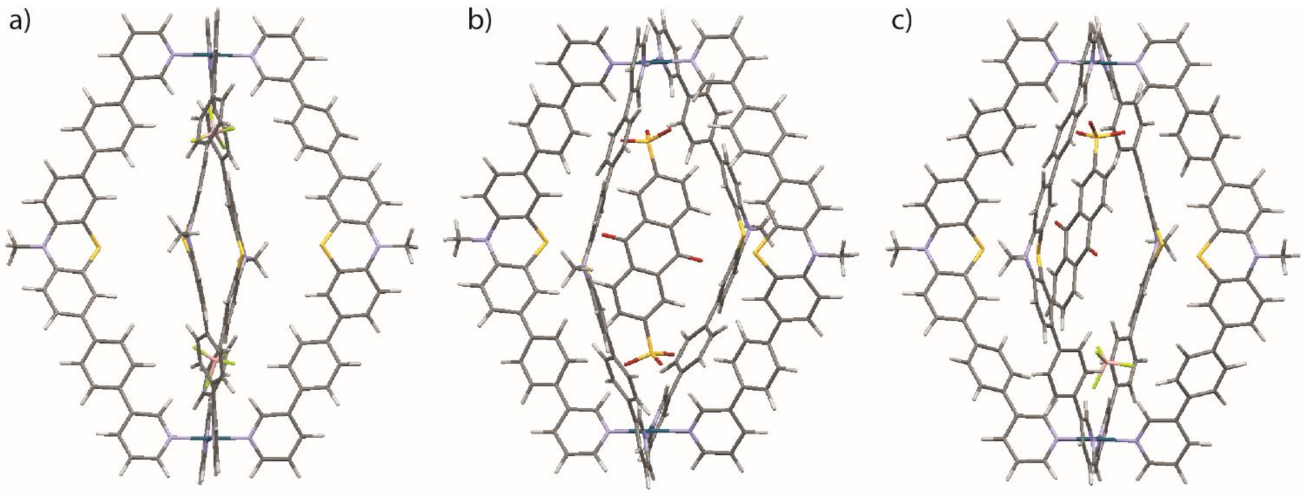

Figure S37: DFT-optimized (B3LYP/def2-SVP) structures: a) [2BF$_4$@Pd$_2$**L**$_4$]$^{2+}$, b) [**G1**@Pd$_2$**L**$_4$]$^{2+}$, and c) [**G3**+BF$_4$@Pd$_2$**L**$_4$]$^{2+}$

4.2 HOMO-LUMO diagrams

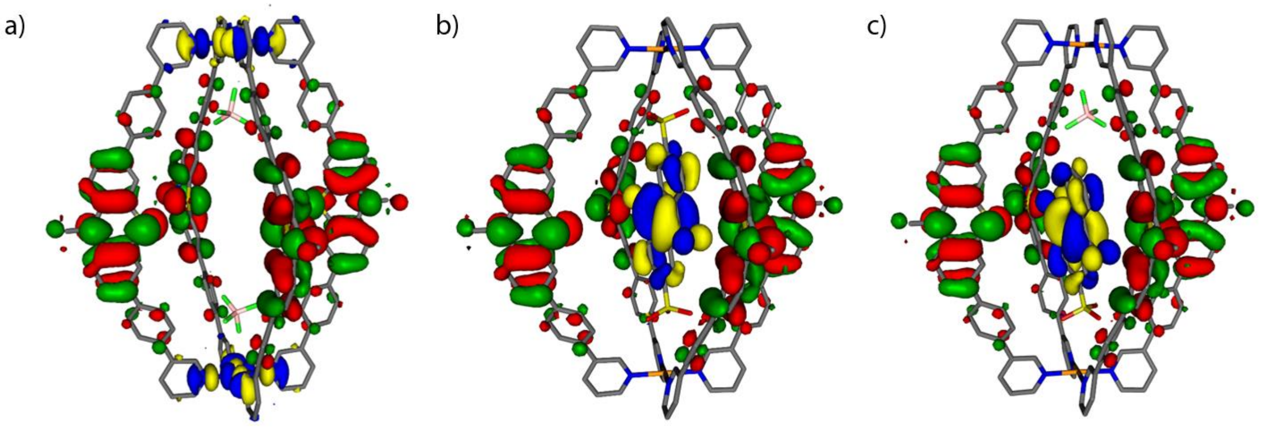

Figure S538: Molecular models with visualization of molecular orbitals. HOMO: red-green, LUMO: yellow-blue. HOMO always fourfold degenerate, LUMO for a) twofold degenerate. a) [**1**+2BF_4]$^{2+}$ b) [**G1@1**]$^{2+}$, and c) [**G3@1**+BF_4]$^{2+}$.

4.3 Table S2. Calculated HOMO-LUMO energies

**Compound**	***E*(HOMO) [eV]**	***E*(LUMO) [eV]**	**ΔE [eV]**	**ΔE [kJ/mol]**	**Orbitals**
[2BF_4@Pd_2**L**$_4$]$^{2+}$	–5.15	–2.23	2.92	281.6	HOMOs spread over PTZ moieties of **L**, LUMOs at Pd centers
[**G1**@Pd_2**L**$_4$]$^{2+}$	–5.24	–3.30	1.95	187.9	HOMOs spread over PTZ moieties of **L**, LUMO at AQ moiety of **G1**
[**G3**+BF_4@Pd_2**L**$_4$]$^{2+}$	–5.17	–3.20	1.97	190.1	HOMOs spread over PTZ moieties of **L**, LUMO at AQ moiety of **G3**

5) UV-Vis studies

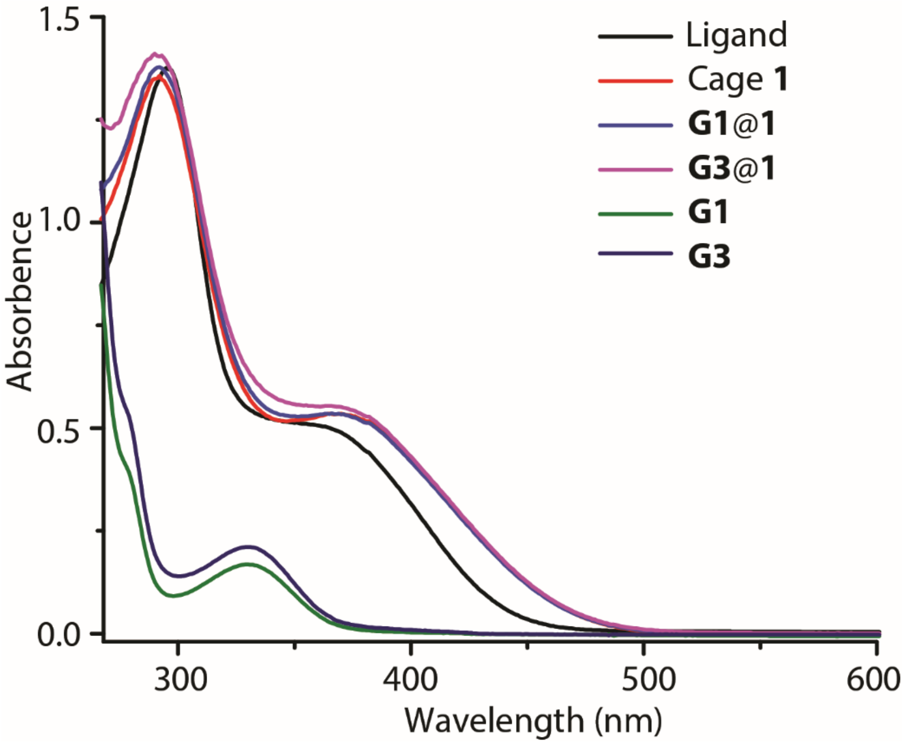

Figure S39: UV-Vis spectra of **L** (0.28 mM), **1** (0.07 mM), **G1@1** (0.07 mM), **G3@1** (0.07 mM), **G1** (0.35 mM) and **G3** (0.35 mM). (Solvent: DMSO; cuvette length: 0.1 cm)

6) Fluorescence studies

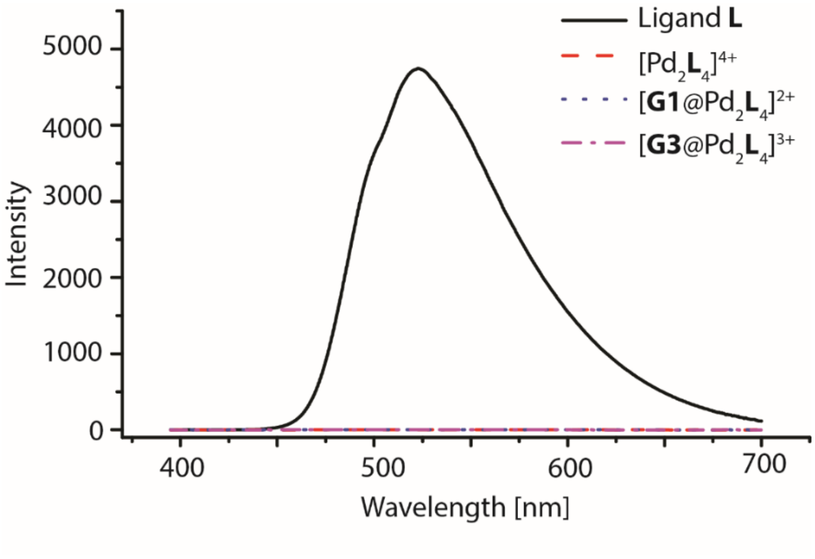

Figure S40: Fluorescence spectra of **L** (0.28 mM), **1** (0.07 mM), **G1@1** (0.07 mM), **G3@1** (0.07 mM), **G1** (0.35 mM) and **G3** (0.35 mM). (The PTZ chromophore concentration in **1**, **G1@1** and **G1@1** is 0.28 mM; Solvent: DMSO; excitation wavelength: 370 nm, emission λ_{max}: 520 nm)

7) Cyclic voltammetry studies

7.1.1 Experimental setup

The cyclic voltammograms were measured with a Metrohm potentiostat PGSTAT101. The data were recorded with the help of the NOVA electrochemistry software (Version 1.9) included in Metrohm Autolab. A homemade electrochemical cell with minimum 3 mL sample volume was used for the electrochemical measurements in a glove box. The sample and electrolyte salt were dissolved in dry and degassed DMSO. The electrochemical cell and degassed samples were arranged in the glove box. A glassy carbon electrode was used as working electrode and a Pt wire as counter electrode. After each measurement, the glassy carbon electrode and the Pt counter electrode were carefully polished with an appropriate polishing powder and rinsed with acetone and DMSO. The minimum sample volume is 3 mL. As reference electrode, the nonaqueous electrode $Ag/AgNO_3$ was utilized. A solution of 0.01 M $AgNO_3$ and 0.1 M tetrabutylammonium perchlorate of electrochemical purity grade was prepared in dry and degassed DMSO and filled inside the electrode. The reference electrode was kept in DMSO/0.1 M tetrabutylammonium hexafluorophosphate solution between the measurement and it was rinsed with DMSO prior to every measurement. All measurements have been performed at room temperature.

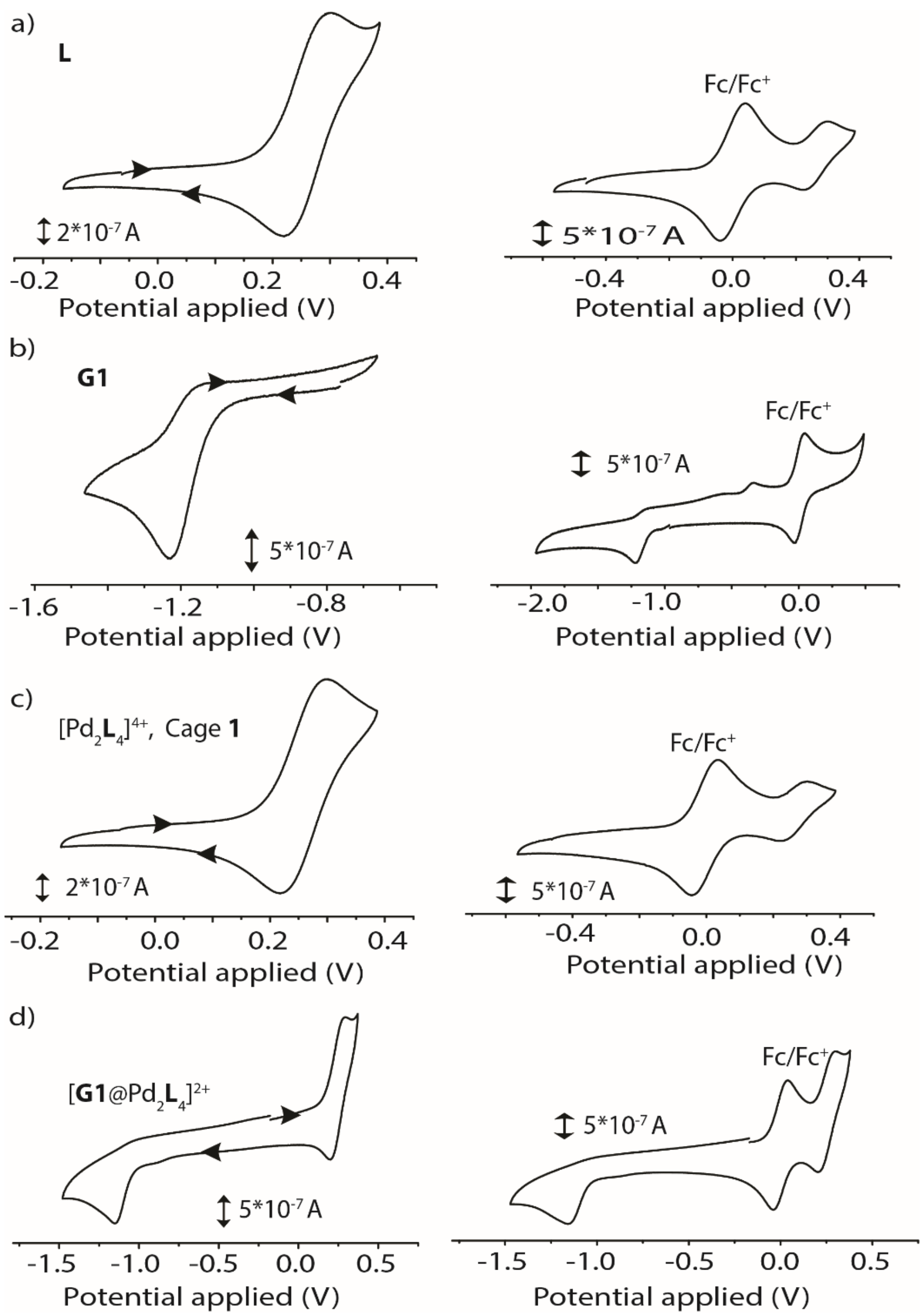

Figure S41: Cyclic voltammograms of samples recorded at a glassy carbon working electrode and Ag/$AgNO_3$ reference electrode of a) Ligand **L** (1.4 mM in DMSO), b) **G1** (0.35 mM in 0.1 M [NBu_4][PF_6] DMSO), c) [Pd_2**L**$_4$]$^{4+}$, cage **1** (0.7 mM in 0.1 M [NBu_4][PF_6] DMSO), d) [**G1**@Pd_2**L**$_4$]$^{2+}$, (0.7 mM in 0.1 M [NBu_4][PF_6] DMSO). The potentials are given with respect to the Fc/Fc$^+$ potential. The CV plots of each sample are shown without (left) or with ferrocene (1 mM, right) as internal reference. Supporting electrolyte: 0.1 M [NBu_4][PF_6]. Scan rate: 0.1 V·s^{-1}.

Table S3: Cyclic voltammetric parameters.

Compound	E_f vs $E_{1/2}$(Fc/Fc$^+$) [V]	E_r vs $E_{1/2}$(Fc/Fc$^+$) [V]	$E_{1/2}$ vs $E_{1/2}$(Fc/Fc$^+$) [V]
Ligand **L**	0.30	0.22	0.26
**G1**	−1.23	−1.17	−1.18
[Pd_2**L_4**]$^{4+}$ Cage **1**	0.30	0.22	0.26
[**G1**@Pd_2**L_4**]$^{2+}$	0.29 (1) and −1.14 (2)	0.20 (1) and −1.08 (2)	0.25 (1) and −1.11 (2)

E_f = forward peak, E_r = reverse peak. (1) first reduction, (2) second reduction step.
Scan rate 0.1 V·s$^{-1.}$

8) Spectroelectrochemistry studies

8.1 Experimental setup

For the spectroelectrochemical measurements a thin layer quartz glass spectroelectrochemical cell was used with an optical path length of 1.0 mm. Instead of a glassy carbon working electrode a Pt mesh electrode was applied. As counter electrode a Pt wire was used. As reference electrode the aforementioned Ag/$AgNO_3$ electrode was used. All spectra were recorded at room temperature. An AvaLight Deuterium-Halogen light source (200 nm - 1000 nm) was used for the UV/VIS measurements. The light was conducted via fiber optic cable (200 µm diameter) to the spectroelectrochemical cell and further to a DAD AVA-SPEC 2048 spectrometer. With every voltage step with a scan rate of 0.1 V/s, the potentiostat PGSTAT101 triggered the measurement of a full UV-Vis spectrum. The data was recorded and processed using the AVASOFT 7.5 software.

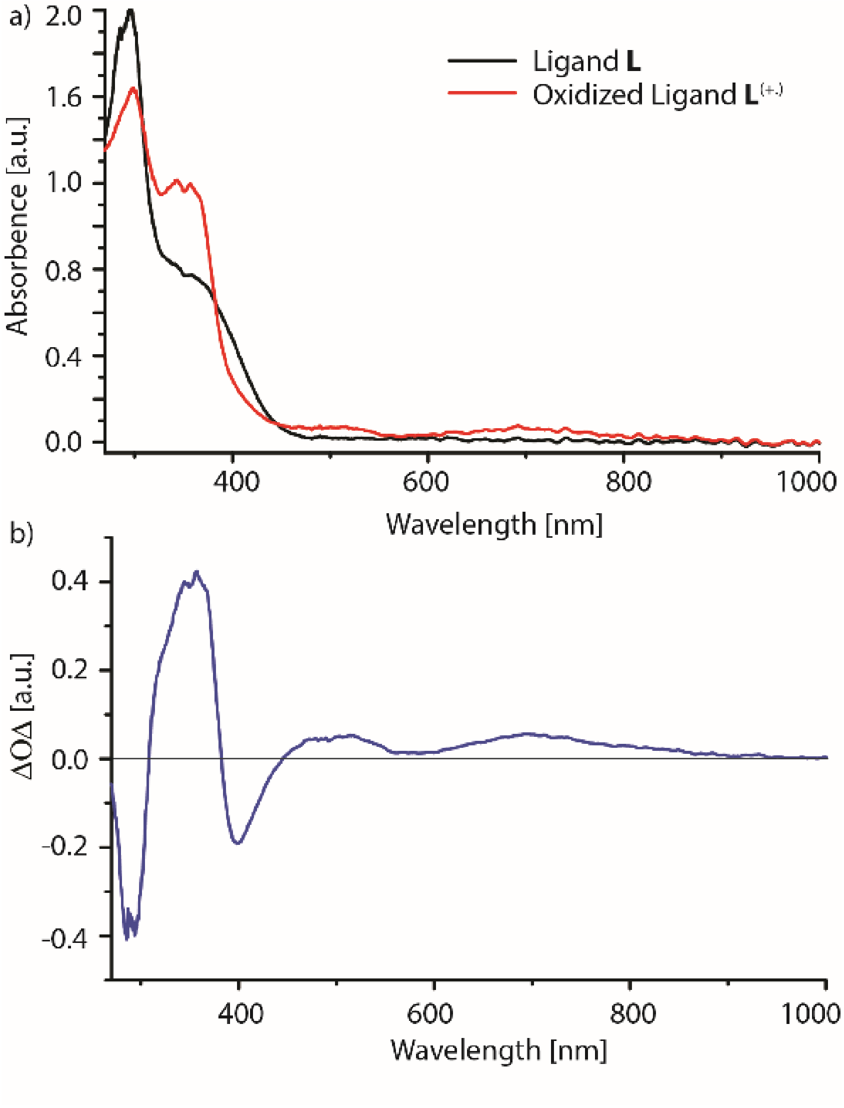

Figure S42: UV-Vis spectra of ligand **L** (1.4 mM in DMSO, 0.1 M NBu_4PF_6) recorded during cyclic voltammetry with a scan rate of 0.1 $V{\cdot}s^{-1}$, Pt gauze as working electrode and $Ag/AgNO_3$ as reference electrode. UV-Vis spectra of a) Ligand **L**, and oxidized ligand and b) Optical difference spectra (compared to initial UV-Vis spectrum of the ligand) of the oxidized ligand.

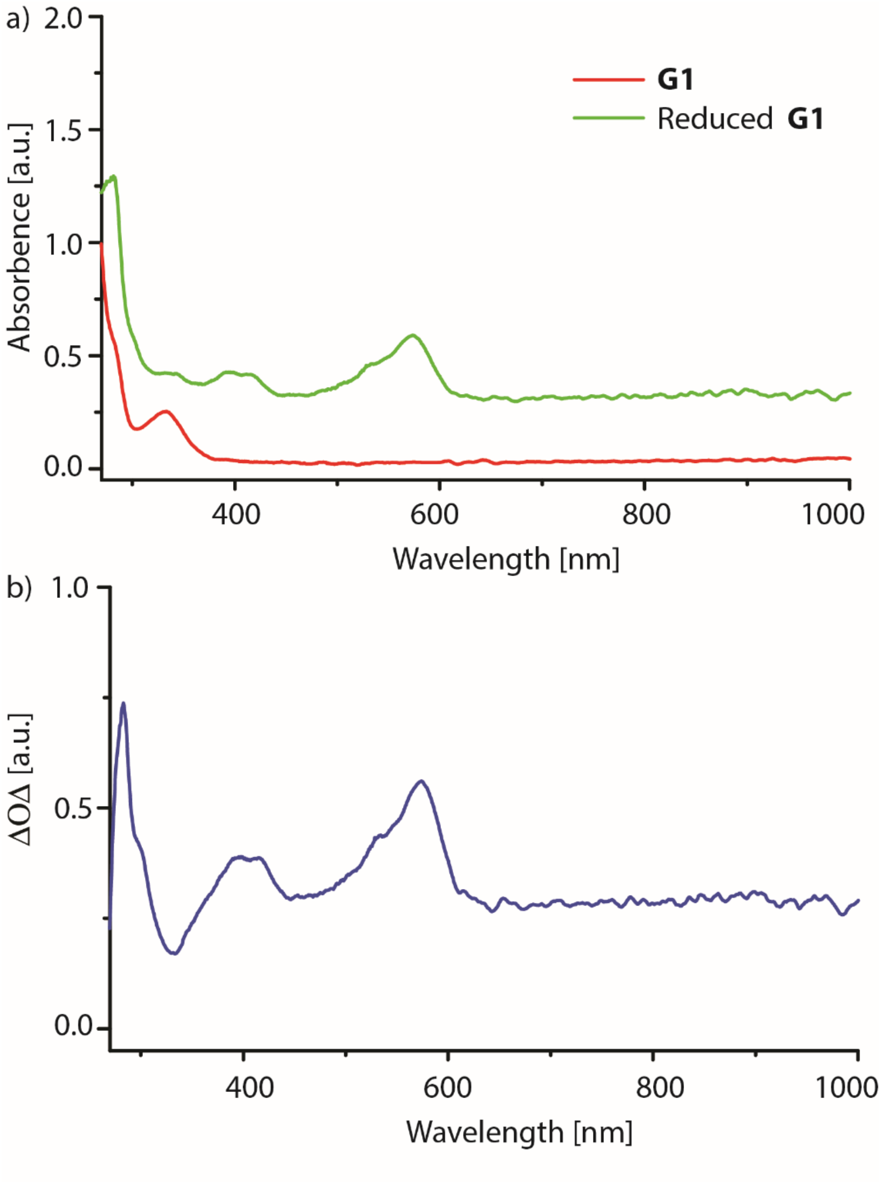

Figure S43: UV-Vis spectra of **G1** (0.35 mM in DMSO, 0.1 M NBu_4PF_6) recorded during cyclic voltammetry with a scan rate of 0.1 $V \cdot s^{-1}$, Pt gauze as working electrode and $Ag/AgNO_3$ as reference electrode. UV-Vis spectra of a) **G1**, reduced **G1** and b) Optical difference spectra (compared to initial UV-Vis spectrum of the **G1**) of the reduced **G1**.

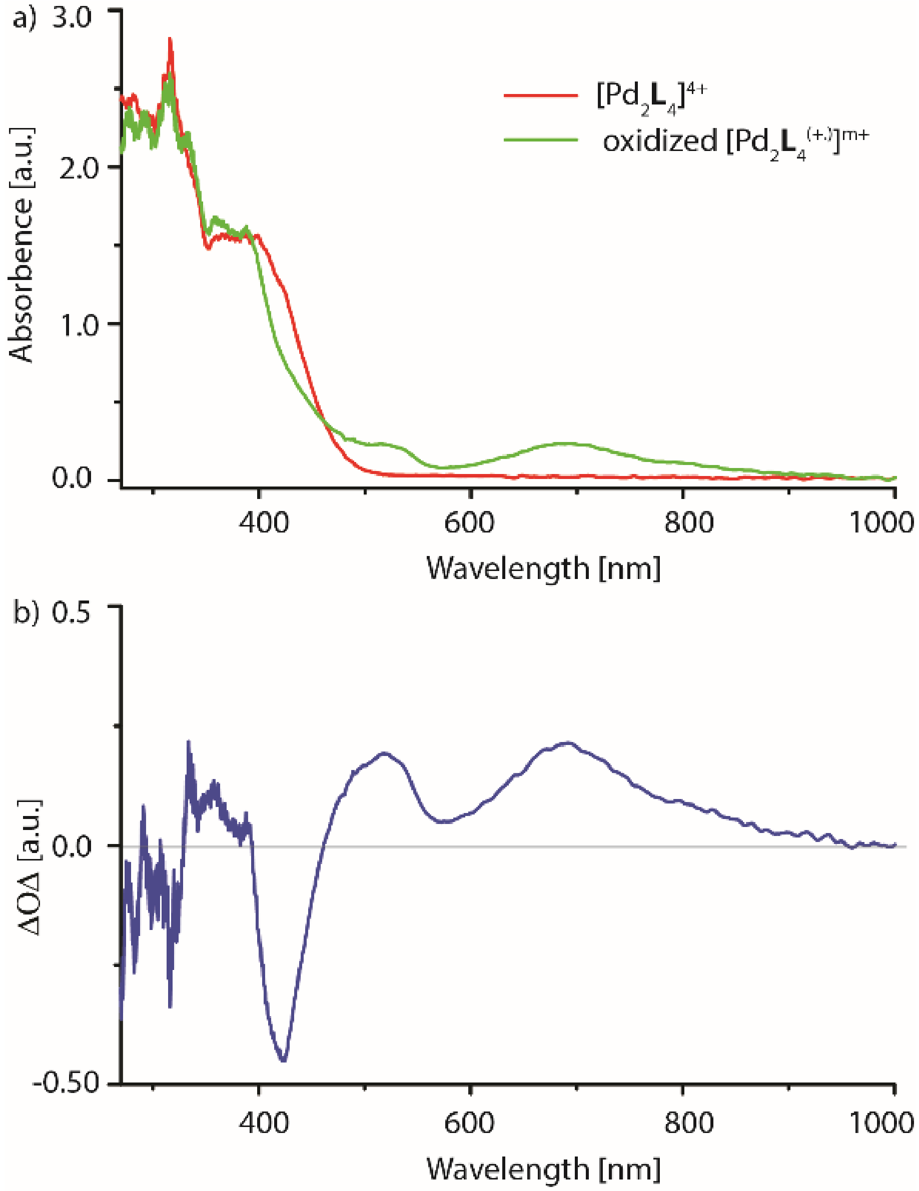

Figure S44: UV-Vis spectra of $[Pd_2\mathbf{L}_4]^{4+}$, cage **1** (0.35 mM in 0.1 M $[NBu_4][PF_6]$ DMSO), recorded during cyclic voltammetry with a scan rate of 0.1 $V{\cdot}s^{-1}$, Pt gauze as working electrode and $Ag/AgNO_3$ as reference electrode. UV-Vis spectra of a) cage **1**, and oxidized cage **1** and b) Optical difference spectra (compared to initial UV-Vis spectrum of the cage **1**) of the oxidized cage **1**.

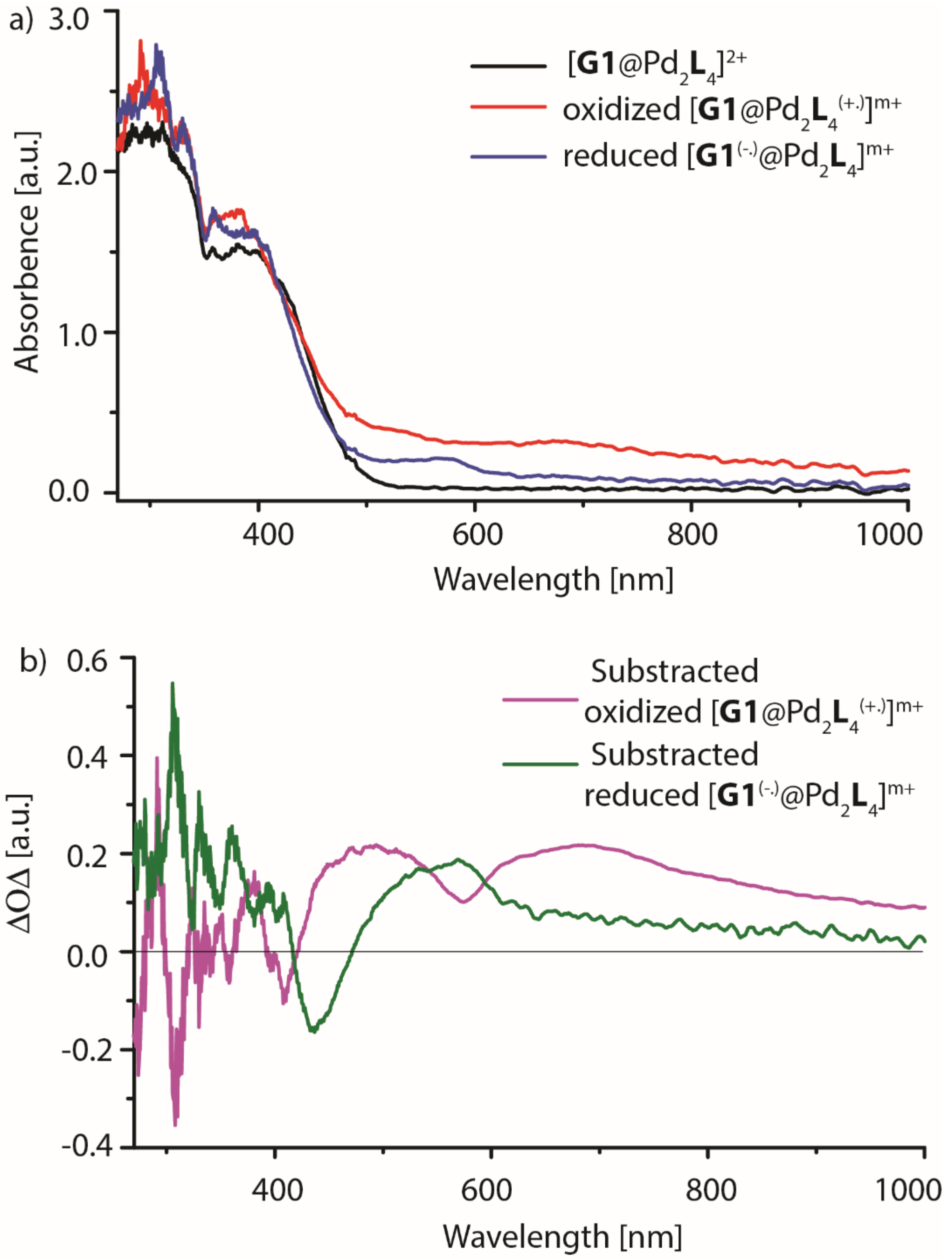

Figure S45: UV-Vis spectra of [**G1**@Pd_2**L**$_4$]$^{2+}$ (0.35 mM in DMSO, 0.1 M NBu_4PF_6) recorded during cyclic voltammetry with a scan rate of 0.1 0.1 V·s^{-1}, Pt gauze as working electrode and Ag/$AgNO_3$ as reference electrode. UV-Vis spectra of a) [**G1**@Pd_2**L**$_4$]$^{2+}$, oxidized and reduced [**G1**@Pd_2**L**$_4$]$^{2+}$, and b) Optical difference spectra (compared to initial UV-Vis spectrum of the ligand) of the oxidized and reduced [**G1**@Pd_2**L**$_4$]$^{2+}$.

.

9) Transient absorption studies

9.1 Transient UV-Vis spectra

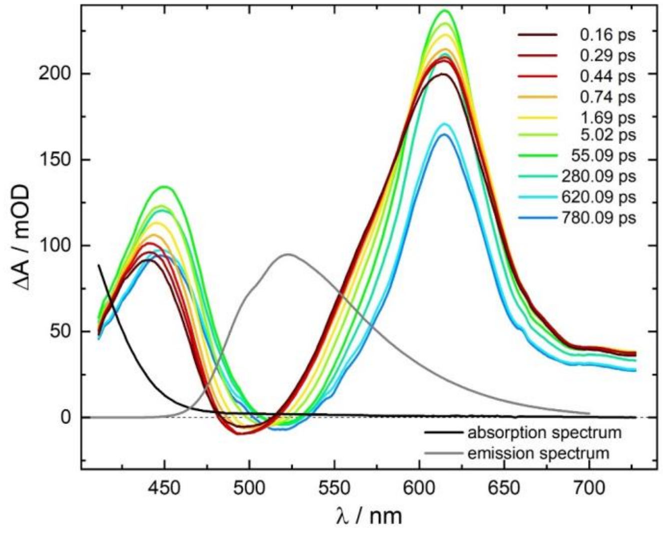

Figure S46: Transient absorption spectra of the free ligand **L** in DMSO following 400 nm excitation; stationary absorption and emission spectra are shown for comparison.

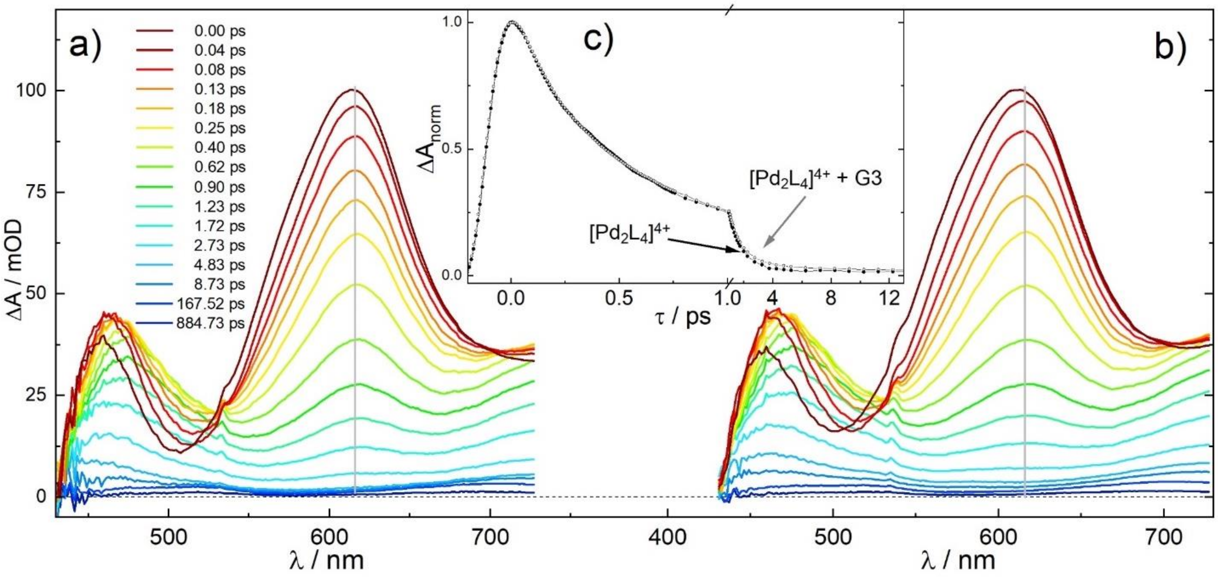

Figure S47: Transient absorption spectra of (a) the bare host cage $[Pd_2\mathbf{L}_4]^{4+}$ at 0.09 mM concentration and (b) in the presence of equimolar amounts of monoanionic guest **G3** (solvent DMSO, pump wavelength 400 nm). The inset c) shows no influence of **G3** on the time traces at the probe wavelength of 613 nm (note that the scaling of time axis changes at 1.0 ps). The reason for this is, that **G3** only features a low binding constant of about 140 M^{-1}, not leading to the formation of significant amounts of the host-guest complex at these low concentrations while **G1** binds stronger ($K \approx 900\ M^{-1}$) and hence leads to population of the host-guest complex even at low concentrations, resulting in the observed charge-transfer phenomena discussed in the main text (compare Fig. S49).

9.2 Kinetic model

The reaction scheme of Figure 8 in the main text results in four coupled differential equations for the relative concentrations in the ground state G, the S_1 state of photo-excited PTZ $\boldsymbol{L}^{S_1}$, the LMCT state, and the HGCT state.

$$\frac{d[\boldsymbol{L}^{S_1}]}{dt} = -(k_{LM} + k_{HG})[S_1]$$

$$\frac{d[LMCT]}{dt} = k_{LM}[S_1] - k_{Pd}[LMCT]$$

$$\frac{d[HGCT]}{dt} = k_{HG}[S_1] - k_{G1}[HGCT]$$

$$\frac{d[G]}{dt} = k_{Pd}[LMCT] + k_{G1}[HGCT]$$

The set of equations was solved numerically with the initial concentration $[\boldsymbol{L}^{S_1}]_{t=0} = 1$ and the other set to zero, yielding their time dependent concentrations. Apart from $\boldsymbol{L}^{S_1}$ there are the spectrally visible species **L**$^{(\bullet+)}$ and **G1**$^{(\bullet-)}$. Their concentration corresponds to $\left[\boldsymbol{L}^{(\bullet+)}\right] = [LMCT] + [HGCT]$ and $\left[\boldsymbol{G1}^{(\bullet-)}\right] = [HGCT]$. To determine the rate constants, we fitted the kinetic model to experimental absorption-time traces $\Delta A(\lambda, t)$ at probe wavelength 474, 505, 613, and 720 nm by summing over all spectrally visible species concentration c_i multiplied by a corresponding relative absorption cross section

$$\Delta A(\lambda, t) = \sum_i \sigma(i, \lambda)\, c_i(t)$$

Fitting first the kinetic traces for the [Pd$_2$**L**$_4$] cage (Figure S48a) yields $k_{LM} = (0.7 \pm 0.1\, ps)^{-1}$ and $k_{Pd} = (0.9 \pm 0.2\, ps)^{-1}$ and relative absorption cross sections for $\boldsymbol{L}^{S_1}$ and $\boldsymbol{L}^{(\bullet+)}$. These were subsequently used without change to fit the time traces for **G1**@[Pd$_2$**L**$_4$] (see Figure 48b) and determine $k_{HG} = (0.9 \pm 0.2\, ps)^{-1}$, $k_{G1} = (2.5 \pm 0.3\, ps)^{-1}$ and the relative cross section of **G1**$^{(\bullet-)}$. The $\sigma(i, \lambda)$ values for $\boldsymbol{L}^{S_1}$, $\boldsymbol{L}^{(\bullet+)}$, and **G1**$^{(\bullet-)}$ summarized in Table S4 are in fair agreement with the spectra shown in Figure S45, and Figure 6 of the main paper, respectively.

Table S4. Relative wavelenght-dependent absorption cross sections derived from the kinetic model

λ / nm	$\boldsymbol{L}^{S_1}$	$\boldsymbol{L}^{(\bullet+)}$	$\boldsymbol{G1}^{(\bullet-)}$
474	29	55	5
505	23	39	16
613	80	8	43
720	34	35	24

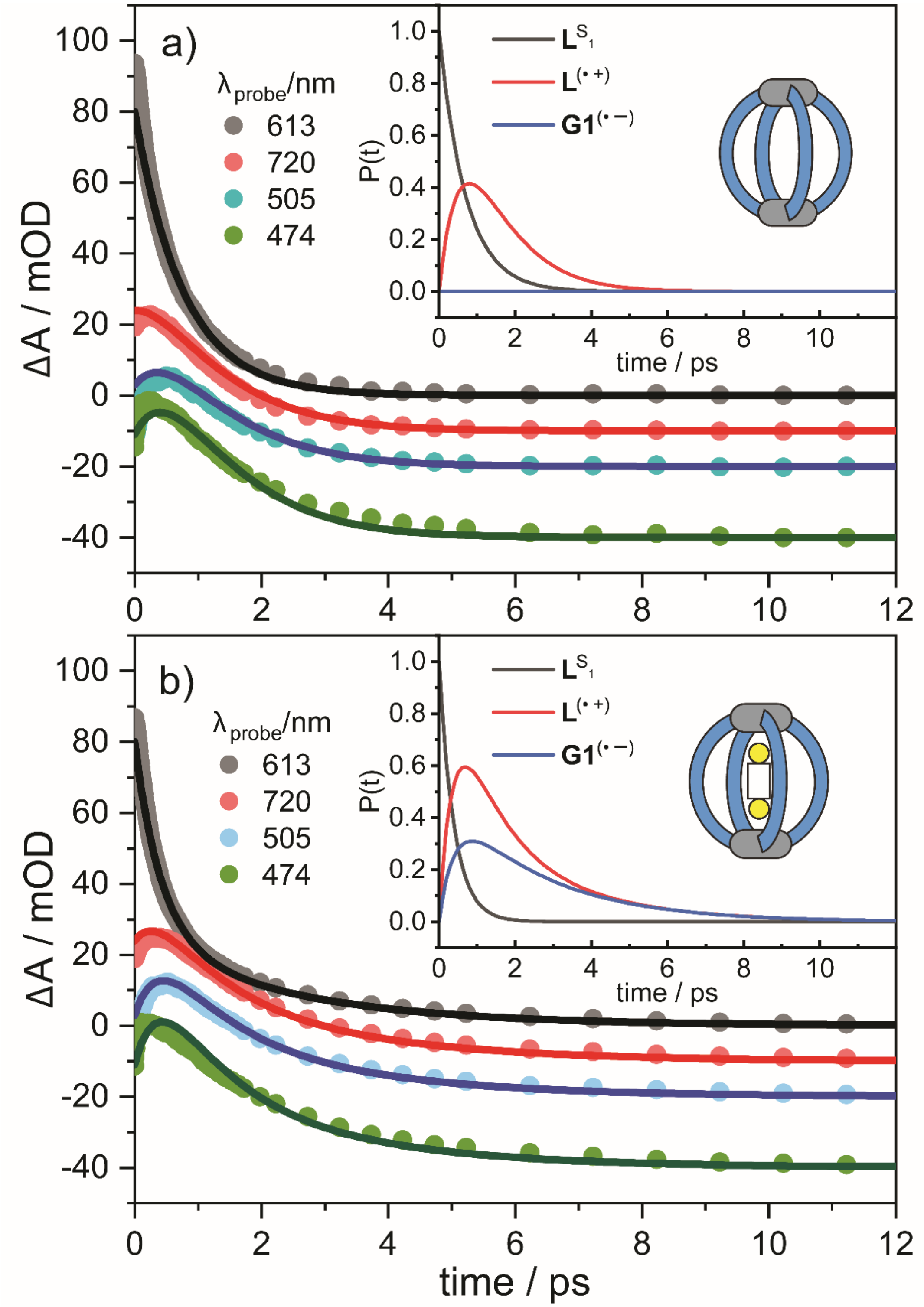

Figure S48: Fits of the kinetic model (lines) to experimental time traces (points) at four selected probe wavelengths for (a) the bare host cage $[Pd_2\mathbf{L}_4]^{4+}$ and (b) in the presence of **G1**. The inserts show the relative populations of $\boldsymbol{L}^{S_1}$, $\boldsymbol{L}^{(\bullet+)}$, and $\boldsymbol{G1}^{(\bullet-)}$.

10) ^{1}H NMR spectra at lower concentration

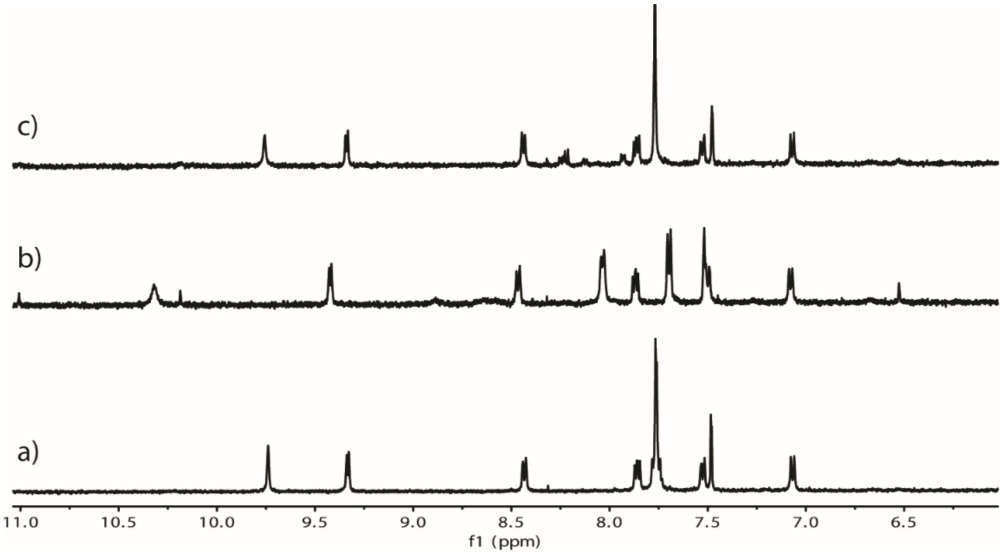

Figure S49: ^{1}H NMR spectra (500 MHz, 298K, DMSO-d_6) of a) cage **1** (0.09 mM), b) [**G1@1**], (0.09 mM), and c) **1** + **G3**, showing that no significant amount of host-guest complex is formed in the latter case at these concentrations (being similar to the ones used in the optical spectroscopy experiments), owing to the low binding constant of monoanionic guest **G3** as compared to **G1** (0.09 mM).

11) References

1. F. Schneck, J. Ahrens, M. Finger, A. C. Stückl, C. Würtele, D. Schwarzer, S. Schneider, *Nat. Commun.*, **2018**, *9*, 1161.
2. B. Schluschaß, J.-H. Borter, S. Rupp, S. Demeshko, C. Herwig, C. Limberg, N. A. Maciulis, J. Schneider, C. Würtele, V. Krewald, D. Schwarzer, S. Schneider, *JACS Au*, **2021**, *1*, 879.
3. M. Frank, J. Hey, I. Balcioglu, Y. Chen, D. Stalke, T. Suenobu, S. Fukuzumi, H. Frauendorf, G. H. Clever, *Angew. Chem. Int. Ed.* **2013**, *52*, 10102.
4. D. B. Hibbert, P. Thordarson, *Chem. Commun.*, **2016**, 52, 12792.
5. O. Plietzsch, A. Schade, A. Hafner, J. Huuskonen, K. Rissanen, M. Nieger, T. Muller, S. Bräse, *Eur. J. Org. Chem.*, **2012**, *2013*, 283.
6. J. B. Grimm, L. D. Lavis, *Org. Lett.*, **2011**, *13*, 6354.
7. T. Gruene, J. T. C. Wennmacher, C. Zaubitzer, J. J. Holstein, J. Heidler, A. Fecteau-Lefebvre, S. D. Carlo, E. Müller, K. N. Goldie, I. Regeni, T. Li, G. Santiso-Quinones, G. Steinfeld, S. Handschin, E. van Genderen, J. A. van Bokhoven, G. H. Clever, R. Pantelic, *Angew. Chem. Int. Ed.* **2018**, *57*, 16313.
8. A. Jerschow, N. Müller, *J. Magnetic Reson. Ser.*, **1996**, *123*, 222.
9. A. Jerschow, N. Müller, *J. Magn. Reson.* **1998**, *132*, 13.
10. E. O. Stejskal, J. E. Tanner, *J. Chem. Phys.*, **1965**, *42*, 288.
11. A. Macchioni, G. Ciancaleoni, C. Zuccaccia, D. Zuccaccia, *Chem. Soc. Rev.*, **2007**, *37*, 479.
12. L. Avram, Y. Cohen, *Chem. Soc. Rev.*, **2014**, *44*, 586.
13. A. Burkhardt, T. Pakendorf, B. Reime, J. Meyer, P. Fischer, N. Stübe, S. Panneerselvam, O. Lorbeer, K. Stachnik, M. Warmer, P. Rödig, D. Göries, A. Meents, *European Phys. J. Plus*, **2016**, *131*, 56.
14. F. Neese, *Wiley Interdiscip. Rev. Comput. Mol. Sci.*, **2018**, *8*:e1327.
